# Tensor-Hypercontracted MP2 First Derivatives: Runtime and Memory Efficient Computation of Hyperfine Coupling Constants

**DOI:** 10.1021/acs.jctc.2c00118

**Published:** 2022-08-09

**Authors:** Felix H. Bangerter, Michael Glasbrenner, Christian Ochsenfeld

**Affiliations:** † Chair of Theoretical Chemistry, Department of Chemistry, University of Munich (LMU), D-81377 Munich, Germany; ‡ Max Planck Institute for Solid State Research, D-70569 Stuttgart, Germany

## Abstract

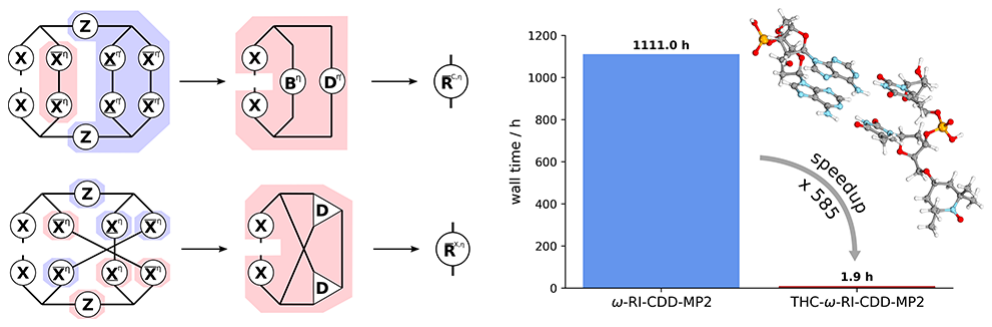

We employ our recently introduced tensor-hypercontracted (THC) second-order Møller–Plesset perturbation theory (MP2) method [Bangerter, F. H., Glasbrenner, M., Ochsenfeld, C. *J. Chem. Theory Comput.***2021**, *17*, 211–221] for the computation of hyperfine coupling constants (HFCCs). The implementation leverages the tensor structure of the THC factorized electron repulsion integrals for an efficient formation of the integral-based intermediates. The computational complexity of the most expensive and formally quintic scaling exchange-like contribution is reduced to effectively subquadratic, by making use of the intrinsic, exponentially decaying coupling between tensor indices through screening based on natural blocking. Overall, this yields an effective subquadratic scaling with a low prefactor for the presented THC-based AO-MP2 method for the computation of isotropic HFCCs on DNA fragments with up to 500 atoms and 5000 basis functions. Furthermore, the implementation achieves considerable speedups with up to a factor of roughly 600–1000 compared to previous implementations [Vogler, S., Ludwig, M., Maurer, M., Ochsenfeld, C. *J. Chem. Phys.***2017**, *147*, 024101] for medium-sized organic radicals, while also significantly reducing storage requirements.

## 1 Introduction

Ever since the advent of modern computers in the 1990s, Møller–Plesset perturbation theory (MP*n*)^1^ has been a good compromise in the family of quantum chemical methods, being sufficiently accurate for many applications while still being computationally affordable.^2^ As opposed to coupled cluster theory (CC), MP*n* lacks infinite-order corrections present in the cluster operator expansion of the CC models, which generally makes MP*n* less accurate.^2^ However, when going from energies to gradients and molecular properties, MP*n*, especially second-order MP*n* (MP2), was shown to yield accurate hyperfine coupling constants (HFCCs)^3−7^ and relative nuclear magnetic resonance (NMR) shifts.^8−12^ However, MP2 is sensitive to spin-contamination in the Hartree–Fock wave function, which can be improved upon when used in its orbital-optimized variant^4^ or as part of double-hybrid density functionals.^13^

Furthermore, when comparing MP*n* and CC at the same expansion orders, for example, MP2 and singles and doubles CC (CCSD), MP*n* comes with a scaling advantage, both in the prefactor and the scaling exponent. Nonetheless, canonical MP2, as well as the associated first and second derivative,^6,11^ still scale with the fifth power of the molecule size, thereby severely restricting the accessible chemical space. To alleviate this limitation several formulations of the MP2 derivatives have been proposed.

Early work from Pulay and Sæbø^14,15^ on local correlation was applied to the computation of MP2 gradients.^16,17^ Following up on this, in recent years the domain-based local pair natural orbital (DLPNO) formulation of MP2 by Neese and co-workers^18−20^ was extended to first and second derivatives. Conceptually related is the divide-expand-consolidate (DEC) formulation of MP2 by Jørgensen and co-workers,^21,22^ which likewise was extended to the computation of molecular gradients in a linear-scaling and massively parallel manner.^23,24^ Instead of exploiting locality in the correlation space, the equations of the MP2 first^6,25^ and second derivative^11^ can be reformulated entirely in terms of atomic orbitals (AOs). However, for an efficient implementation and the reduction of the scaling prefactor, an orbital localization by pivoted Cholesky decomposition (PCD) of the associated pseudodensities is essential.^6^

Besides ensuring low-scaling and efficiency, for derivative calculations of electron correlation methods in general, it is pivotal to efficiently manage the available memory and disk space. Compared to the energy equations, the associated gradients and higher derivatives often not only include electron repulsion integrals (ERIs), in either atomic or molecular orbital basis, but can also include partially transformed ERIs and derivatives thereof, for example, with respect to the magnetic field in NMR calculations. Since canonical ERIs are fourth-order tensors, their memory requirements prohibitively scale with the forth power of the number of basis functions and saving multiple such tensors quickly becomes unfeasible. To reduce the memory footprint of the ERIs, tensor decomposition methods, particularly the resolution-of-the-identity (RI) ansatz,^26−29^ have been broadly applied in the context of MP2 derivatives.^5,6,12,30^ However, with increasing molecule size, even the third-order RI tensors eventually exceed the available disk space of conventional high performance computing nodes. To overcome this storage limitation, further reduction of the dimensionality of the ERIs is desirable. This can be achieved by the recently introduced tensor hypercontraction (THC) factorization of Martínez and co-workers,^31−34^ which in general terms approximates a fourth-order integral tensor (*μν*|*ô*|*λσ*), where *ô* is a singular two electron interaction kernel, by five second-order tensors.^35^ In the least-squares formulation of THC (LS-THC),^31^ four of these tensors are simply obtained by evaluation of the basis functions at real-space grid nodes and the singular *ô* operator is replaced by the LS-fitted **Z** matrix. If *ô* is the Coulombic 1/*r* operator, a factorization of the regular ERIs is achieved, which has been employed in reduced scaling formulations of exact exchange,^36^ different orders of MP*n*,^31,33−41^ the random phase approximation (RPA),^42^ complete active space perturbation theory (CASPT2),^43^ and various flavors of CC theory,^44−46^ as well as equation-of-motion CC (EOM-CC) theory.^47^ Recently Matthews^35^ thoroughly investigated amplitude factorizations within MP3, as a stepping stone toward CCSD, and noted that the LS-THC factorization of nonlocal integrals, such as the exchange integrals, incurs an additional error.

The applicability of THC for the computation of molecular gradients is largely unexplored. Song et al.^39^ derived equations for the analytical gradient of THC-AO-MP2 with application to geometry optimizations and *ab initio* molecular dynamics (AIMD) simulations. As commonly the derivation of gradient equations of electron correlation methods is rather involved, Song et al.^48^ also proposed an automatic differentiation scheme for the automated generation of working equations for gradients of THC-based correlation methods.

When it comes to applying tensor factorizations, be it RI or THC, two possible routes to molecular gradients can be taken: The first approach is to differentiate the RI- or THC-approximated energy equation, then the associated gradient describes the slope of an approximated potential energy surface (PES). The second approach is to take the gradient of the canonical energy and insert the approximation into the exact gradient; this way an approximate gradient is used to move along the exact PES.

The latter can lead to an unwanted buildup of errors during the course of a simulation, when used in molecular dynamics simulations. However, when used in conjunction with thermostats constant energy is traded for constant temperature, and depending on the extent of the gradient error, this approach can still be applicable but must be tested for the chosen gradient. The first approach was taken by Song et al.^39^ for their THC-based MP2 molecular gradient, whereas in the present work, the gradient equations with respect to a perturbation, that the basis functions are independent of, are derived by inserting the THC factorization into the equations of the exact gradient. As will be discussed in section 2.4, the resulting equations are identical to the ones obtained by differentiating the THC-AO-MP2 energy equation. As a representative case of these kinds of perturbations, we apply our recently developed low-scaling LS-THC algorithm^41^ to the AO-MP2 energy derivative with respect to the nuclear magnetic moment, for the computation of HFCCs on the MP2 level of theory.

We present ways to efficiently treat the Coulomb- and the exchange-like part of the most expensive intermediate of the derivative within the LS-THC approximation. We demonstrate the low-scaling behavior of our THC-ω-RI-CDD-MP2 derivative method for various chemically relevant systems. We also show that THC-ω-RI-CDD-MP2 significantly outperforms our previous implementation of ω-RI-CDD-MP2^6,30^ for the computation of isotropic HFCCs.

## 2 Theory

### 2.1 Notation

Throughout this publication we make use of the following indices:

- μ, ν, λ, σ: atomic orbital indices belonging to the AO basis {χ_μ_} of size *N*_bf_.
- α, β, γ, δ: auxiliary basis indices belonging to the density fitting basis {χ_α_} of size *N*_aux_ (usually *N*_aux_ ≈ 3·*N*_bf_).
- *P*, *Q*, *R*, *S*: grid point indices belonging to the LS-THC grid of size *N*_grid_ (usually *N*_grid_ ≈ 3·*N*_aux_).
- *i*, *j*: occupied molecular orbital indices belonging to the MO basis {ϕ_*i*_} of size *N*_occ_.
- *a*, *b*: virtual molecular orbital indices belonging to the MO basis {ϕ_*a*_} of size *N*_virt_ (*N*_virt_ ≫ *N*_occ_).
- η, η′: spin indices, for either α- or β-electrons, with η′ ≠ η.
- κ: index of the Laplace quadrature points for the MP2 energy denominator with weights ω_κ_ (usually integration with 5–8 points is sufficiently accurate).
- *k*: index of the nucleus under consideration.

### 2.2 Review of the AO-MP2 Gradient

The unrestricted AO-MP2 energy with a Laplace transformation^49−51^ for the energy denominator is given as
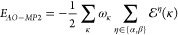
1with
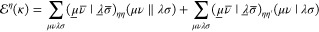
2for which the transformed ERIs are given by
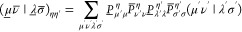
3and **P̲** and **P̅** are the usual pseudodensities, given by
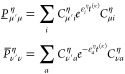
4

To obtain the AO-MP2 gradient, eq 1 has to be differentiated with respect to a perturbation ξ. Since the focus of this work is on HFCCs, as an example for a property for which the basis functions are independent of the perturbation—here ξ′—the following derivation is restricted to this special case. An in-depth derivation of the AO-MP2 gradient equations, as well as a comparison to the MO-MP2 gradient, is available in refs (6) and (25).

In order to obtain the gradient of the AO-MP2 energy, eq 1 is differentiated with respect to ξ′
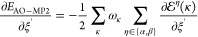
5with
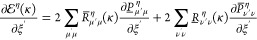
6and intermediates **R̲** and **R̅** given by
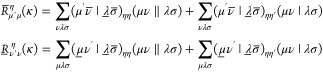
7The above **R** intermediates can be thought of as the contraction of all perturbation-independent parts of the gradient. Note that eq 5 only involves the derivative of the pseudodensities and no integral derivatives, as the basis functions are taken to be independent of ξ′. In order to avoid the evaluation of the derivatives of the perturbed occupied and virtual pseudodensities, these intermediates are expanded in terms of the regular occupied and virtual densities **P**_occ_ and **P**_virt_ as
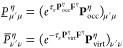
8Thus, the derivatives of eq 8 are given by
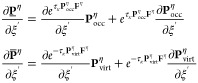
9By further making use of the identity
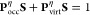
10the perturbed virtual density can be related to the perturbed occupied density as
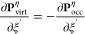
11Note again that eq 11 does not contain the derivative of the overlap matrix **S**, due to **S** being independent of ξ′. By making use of the above relations for the densities, eq 6 becomes
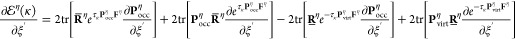
12Cyclic permutation under the trace was applied above to obtain terms of the general form
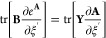
13with
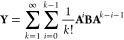
14which can be solved for **Y** by recursion, as detailed in refs (25) and (12). Let **Y̅**^η^ be the solution of eq 13 with **A** ≡ τ_κ_**P**_occ_^η^**F**^η^ and **B** ≡ **P**_occ_^η^**R̅**^η^, and let **Y̲**^η^ be the solution with **A** ≡ – τ_κ_**P**_virt_^η^**F**^η^ and **B** ≡ **P**_virt_^η^**R̲**^η^. Then, by making use of the other transformations outlined above, the derivative from eq 6 can be rewritten as
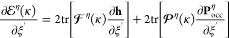
15with
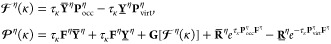
16where intermediate  resembles the Fock matrix with  substituting for the density matrix; that is, 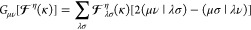
17Note that the first term in eq 15 only includes the derivative of the core Hamiltonian matrix as the integrals in the Fock matrix, from which this term originates from, are independent of ξ′.

Equation 15 permits an elegant solution for the perturbed density, avoiding the need to solve coupled-perturbed self-consistent field (CPSCF) equations for all perturbations ξ′, by means of applying a AO-based *Z*-vector-like method^25^ originally proposed by Handy and Schaefer.^52^ The implicit first derivative of the occupied density can therefore be efficiently obtained by applying the density matrix-based Laplace-transform unrestricted CPSCF (DL-UCPSCF) method by Beer and Ochsenfeld.^53^ The intricacies of this method are detailed in refs (53, 25), and (6).

To conclude, eqs 5 and 15 yield the first derivative of the AO-MP2 energy with respect to a perturbation ξ′. More specifically, if ξ′ was an external electric field then eq 5 would yield permanent dipole moments, and if ξ′ was equal to the nuclear magnetic moment *M*_*k*_ of a given nucleus *k* then the isotropic contribution to the HFCC of nucleus *k* in the absence of spin–orbit coupling would be obtained. The latter property will be used as a sample property for the newly developed THC-ω-RI-CDD-MP2 derivative method presented in section 2.4.

### 2.3 RI-CDD-MP2 HFCCs

The computational bottleneck of obtaining the AO-MP2 gradient in eqs 5 and 15 are the integral contractions in the formation of the **R** matrix intermediates given by eq 7. Because forming the **R**-matrices involves the same contraction with the pseudodensities as the AO-MP2 energy, the computation of the gradient will *a priori* also have quintic scaling. As is common practice when dealing with these kinds of integral contractions, the RI approximation can be inserted into eq 7 to lower the computational cost as well as the memory requirements by avoiding the fourth-order ERI tensors. To further lower the prefactor of the integral transformations a PCD of the pseudodensities can be used, which is known as the Cholesky-decomposed pseudodensity (CDD) approach. The CDD method produces a set of so-called Cholesky pseudo-MO coefficient matrices **L̲** and **L̅** according to
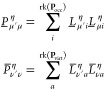
18which are used analogously to the regular MO coefficients. The combined RI-CDD approach leads to a reformulation of the integrals incorporated in the **R** intermediates as
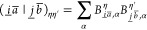
19with
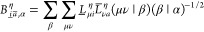
20

In combination with QQR-type integral screening the RI-CDD-MP2 gradient method was shown to yield cubic scaling for the computation of molecular gradients and HFCCs.^6^ To further reduce the scaling, Vogler et al.^30^ employed the attenuated Coulomb metric^54,55^ in the RI approximation as well as the scaled-opposite spin (SOS)^56^ approximation, which removes the same spin contribution entirely. Still, the expensive formation of the **R** intermediates has to be done for every Laplace point and thus constitutes the predominant part of the wall time for the evaluation of the MP2 gradient with respect to a perturbation ξ′, even with the ω-RI-CDD-SOS-MP2 method.^6,30^

### 2.4 THC-CDD-MP2 HFCCs

ERIs are ubiquitous in electron correlation methods and their transformation and contraction usually represents the bottleneck of the calculation. This is especially the case when many different ERIs, that is, fully and partially transformed into the MO space or contracted with a perturbed density matrix, are needed, and their formation has to be carried out repeatedly, such as inside a Laplace expansion or during the iterative solution of amplitude equations. Since the scaling behavior of these operations is dependent on the dimensionality of the representation of the ERI tensor, a most compact representation is desirable. Of particular interest is thus the THC factorization, which in its AO formulation approximates an ERI as
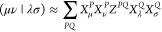
21and—in LS-THC—the **X**-matrices are simply obtained by evaluation of basis functions at the THC grid.^31^ The analytical expression of the **Z**-matrix can be shown to be the solution to the normal equations associated with the least-squares equation of finding the THC factorization (see the Supporting Information). As has been shown previously,^33,34,36,39,41−43^ the THC factorization can achieve major savings in computation time for intermediates involving ERI contractions, by reducing the representation of the ERIs to only second-order tensors. In this work, we make use of our recently reported low-scaling THC method^41^ based on the ω-RI approximation for the ERIs contained in **Z** and natural blocking (NB).^57,58^ However, in contrast to our work on THC-MP2 energies,^41^ in the present work the AO ERIs are fitted, since the gradient for calculating the MP2 HFCCs is based on our RI-CDD approach to the computation of AO-MP2 energy gradients.^6^ It is important to note here, that while the equations for the THC-based gradient method are derived by inserting the THC factorization into the equations of the RI-CDD-MP2 gradient with respect to ξ′, there is no difference to directly differentiating the THC-AO-MP2 energy equations. This is the case, because neither **X** nor **Z** depend on the perturbation ξ′ in the AO-THC formulation. This independence would not be given if either the MO-THC approach was used or for the more general derivative with respect to ξ, which would necessitate the derivatives of the THC tensors. In other words, by simply inserting the THC factorization into eq 7 the derivative of the THC-ω-RI-CDD-MP2 energy with respect to ξ′ is obtained.

To reduce the computation time needed for forming the expensive **R**-matrices of the UMP2 gradient, the AO-THC factorization is inserted into eq 7 in its RI-CDD formulation to yield
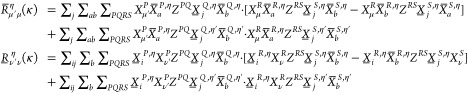
22where **X̲** and **X̅** are the collocation matrices **X** transformed into the occupied and virtual Cholesky pseudo-MO basis, respectively. In MP2 it is often advisable to treat Coulomb- and exchange-like contributions separately,^58^ thus the **R**-matrices are partitioned into **R**^C^, the Coulomb-like contribution, and **R**^X^, the exchange-like contribution.

#### 2.4.1 THC **R**-Matrices: Coulomb-like Contribution

The Coulomb-like parts **R̲**^C^ and **R̅**^C^ are given by
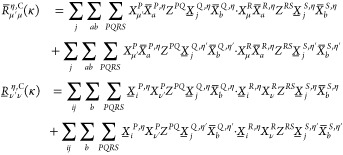
23and can—in analogy to the THC-MP2 energy—be efficiently computed using sparse linear algebra.^41^ In doing so, **R̲**^C^ and **R̅**^C^ are especially efficient to compute, as their formation only involves BLAS level 3 operations. For an efficient implementation it is important to realize, that for large enough molecules and appropriate ordering of the THC grid points, the collocation matrices **X** become sparse, while the **Z**-matrix, being the representation of the long-ranged 1/*r* operator, will always remain dense. By closer inspection of eq 23, it can be noticed, that the **X**-matrices corresponding to the MOs of the ket of the decomposed ERI and the **Z**-matrix are identical for all terms. Therefore, these matrices can be collected in an intermediate **D**^η^ given by Algorithm 1, where intermediates **A** and **B** are given by
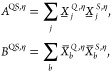
24and represent the occupied and virtual pseudodensity in the grid basis, respectively. Additionally, the **Λ**-factorization of the **Z**-matrix, i.e., **Z** ≡ **ΛΛ**^T^, is used to lower the prefactor of this step.^34,41^
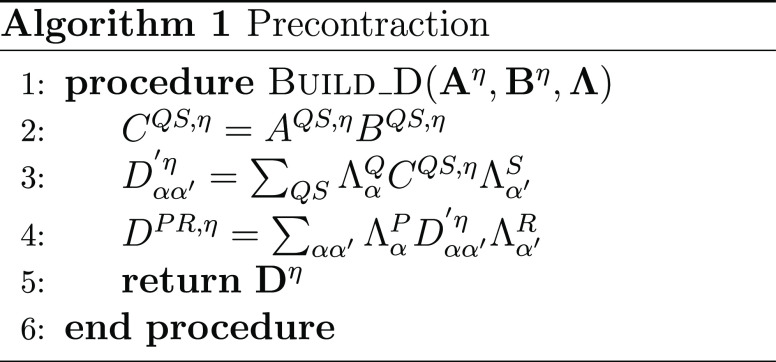


As forming intermediate **D** requires a series of dense matrix–matrix-multiplications, this step will require the majority of the computation time for **R̲**^C^ and **R̅**^C^. However, Algorithm 1 has to be performed only once per Laplace point and electron spin. The final contribution to the **R**-matrices can then simply be obtained by a Schur product and two matrix–matrix-multiplications given by Algorithm 2.
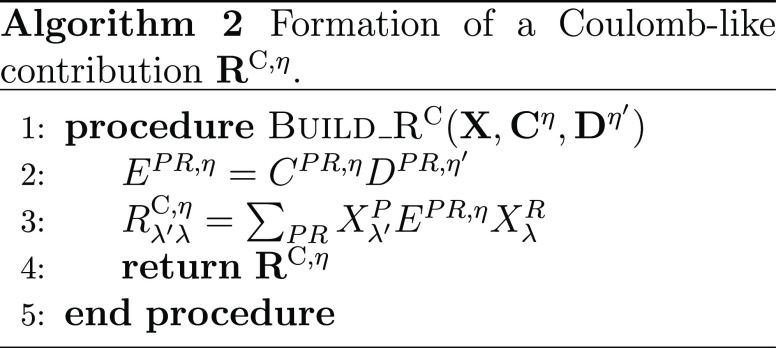


**Figure 1:**
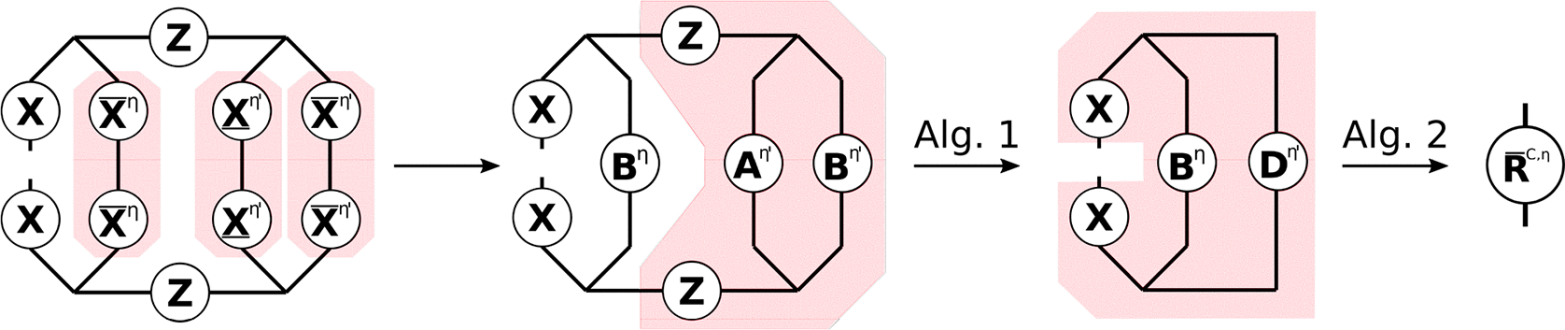
Tensor network representation of the contractions performed by Algorithms 1 and 2 for the formation of a Coulomb-like contribution **R̅**^C,η^. By symmetry, **R̲**^C,η^ can be formed analogously, but with intermediates **A** and **B** interchanged. Tensors contracted in ensuing steps are highlighted.

The effects of Algorithms 1 and 2 can be best understood by visualizing the underlying tensor contractions in a tensor network diagram, as given in Figure 1. For an introduction of tensor network diagrams, also in the context of THC, refer to the work by Schutski et al.^46^ Algorithms 1 and 2 are then pieced together with the algorithm for the exchange-like part, detailed in the next section, for the final Algorithm 4 in section 2.4.3.

#### 2.4.2 THC **R**-Matrices: Exchange-like Contribution

**Figure 2:**
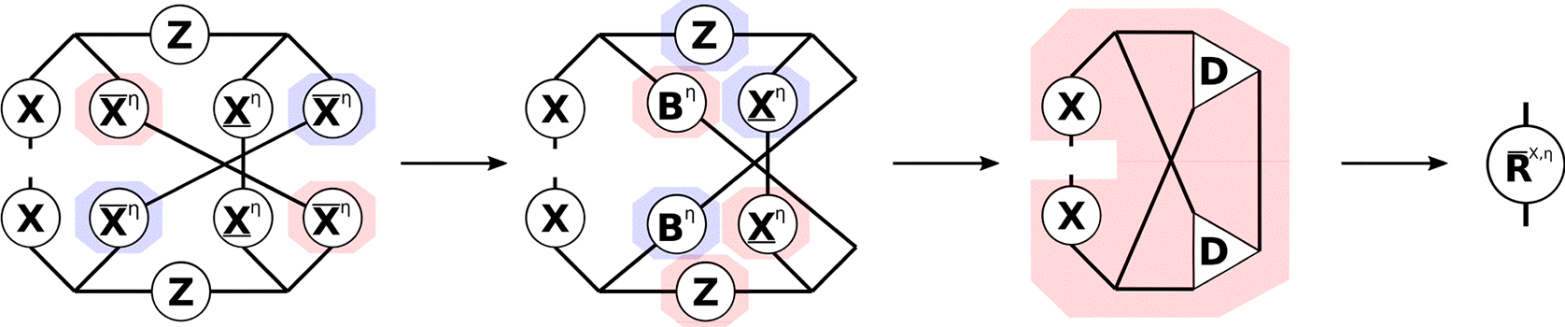
Tensor network representation of the contractions performed by Algorithm 3 for the formation of an exchange-like contribution **R̅**^X,η^. By symmetry, **R̲**^X,η^ can be formed analogously, however, in Algorithm 3 a different approach is used to reduce the prefactor. Tensors contracted in ensuing steps are highlighted.

Usually when higher than second-order tensors arise, the associated tensor contractions are either carried out with the tensors reshaped into matrices or batched over the dimensions exceeding matrix dimensionality in so-called tensor slices. For an efficient contraction when iterating over tensor slices it is advisable to make use of an underlying structure to reduce the dimensions of the slices, either by matrix decomposition of the slice or by neglecting noncontributing elements. If whole rows and columns are excluded based on some significance criterion, one arrives at the natural blocking (NB) formalism^57,58^ for tensor contractions. NB relies on significance lists, which in general terms describe which pairs, of a general index pair *i* and *j*, contribute to a tensor contraction involving these indices. Mathematically speaking these lists are sets and thus can, in set-builder notation, be represented as
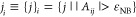
25where **A** is a screening matrix involving indices *i* and *j*, and ε_NB_ is the NB screening threshold. To avoid confusion, we use *j*_*i*_ as a shorthand notation for the set of all significant *j* for a particular index *i* and {*j*_*i*_} for the set of all *j*_*i*_. If two elements in a set {*j*_*i*_} are identical, it technically becomes a multiset, as elements in sets are only allowed to have a multiplicity of 1. In set terminology, the set {*i*}_*j*_ is the transpose of {*j*}_*i*_ and can analogously determined from **A**^T^ as
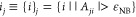
26or directly from {*j*_*i*_}. Another important quantity is the number of significant pairs *N*_*ij*_, which is defined as
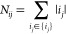
27NB and THC work particularly well together for exchange-like contractions of ERIs, as within the THC formalism the necessary screening matrices can easily be constructed as outlined in the following. The following prototypical exchange-like ERI contraction from the THC-CDD-MP2 energy expression will serve as an example:
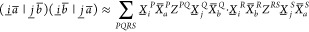
28Two types of indices are present in the above equation, the orbital indices *i* and *j* (occupied space) as well as *a* and *b* (virtual space) and the THC auxiliary indices *P*, *Q*, *R*, and *S*. In the following discussion we use the LS formulation of THC, but the statements also hold for other THC variants. Thus, there are three general types of index pairs: orbital–orbital, orbital–grid, and grid–grid index pairs. The set of significance lists for orbital-grid type pairs is especially easy to construct, since it can be directly derived from the collocation matrices **X**. For example, the set of all significant grid points *P* for a given occupied orbital *i* can be constructed as
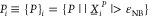
29Significant orbital–orbital pairs are also easily obtained from the collocation matrices; for example, the set of significant virtual orbitals *a* for a given occupied orbital *i* can be built as
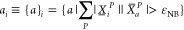
30The screening criterion from eq 30 can also be interpreted to yield only *ia* pairs, for which the orbitals have significant overlap and which produce non-negligible charge densities. For the development of low-scaling exchange-type contractions it is important to make use of the exponential coupling between all orbital indices. Orbital *i* couples to orbital *a* in an exponentially decaying fashion in the bra of the first ERI in eq 28. Likewise, orbital *j* also couples to orbital *a* in an exponentially decaying fashion in the ket of the second ERI. Thus, there is indirect exponential coupling between orbitals *i* and *j* via orbital *a* and the set of significant orbitals *i* for a given orbital *j* can be derived from the sets {*a*_*j*_}, which is identical to {*a*_*i*_}, and {*a*_*i*_}:
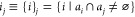
31With the significance lists given by eqs 29, 30, and 31, an asymptotically linear scaling algorithm for the exchange-like energy contribution to MP2 can be devised. The algorithm is detailed in the Supporting Information and close to linear scaling is demonstrated. Here, however, the focus lies on the exchange-like parts of the **R**-matrices, which are conceptually similar, but different in that two AO indices remain uncontracted. The tensor contractions necessary for forming the **R**^X^ parts can again best be understood from the tensor network diagram in Figure 2.

While Figure 2 only shows the **R̅**^X^ part, the **R̲**^X^ contribution can be constructed analogously. First, intermediates **A** or **B** are formed from the collocation matrices **X̲** or **X̅**, respectively. Second, the remaining collocation matrices, the **Z**-matrices and intermediates **A** or **B** are contracted to the third-order tensor intermediate **D**, where the symmetry of the tensors is used to reduce the operation count. The idea for an efficient implementation is then as follows: to avoid storing third-order tensors, the contraction is batched over the occupied orbital index common to both **R̲** and **R̅**, and to reduce the cost of the dgemm operations within the loop NB is applied. In this way separate algorithms for **R̲**^X^ and **R̅**^X^ can be formulated, which are presented in the Supporting Information. By closer inspection, however, it can be seen, that both **R**^X^-matrices share the most expensive to compute intermediate, which incorporates the **Z**-matrix. Therefore, a joint computation as given by Algorithm 3 is preferred.
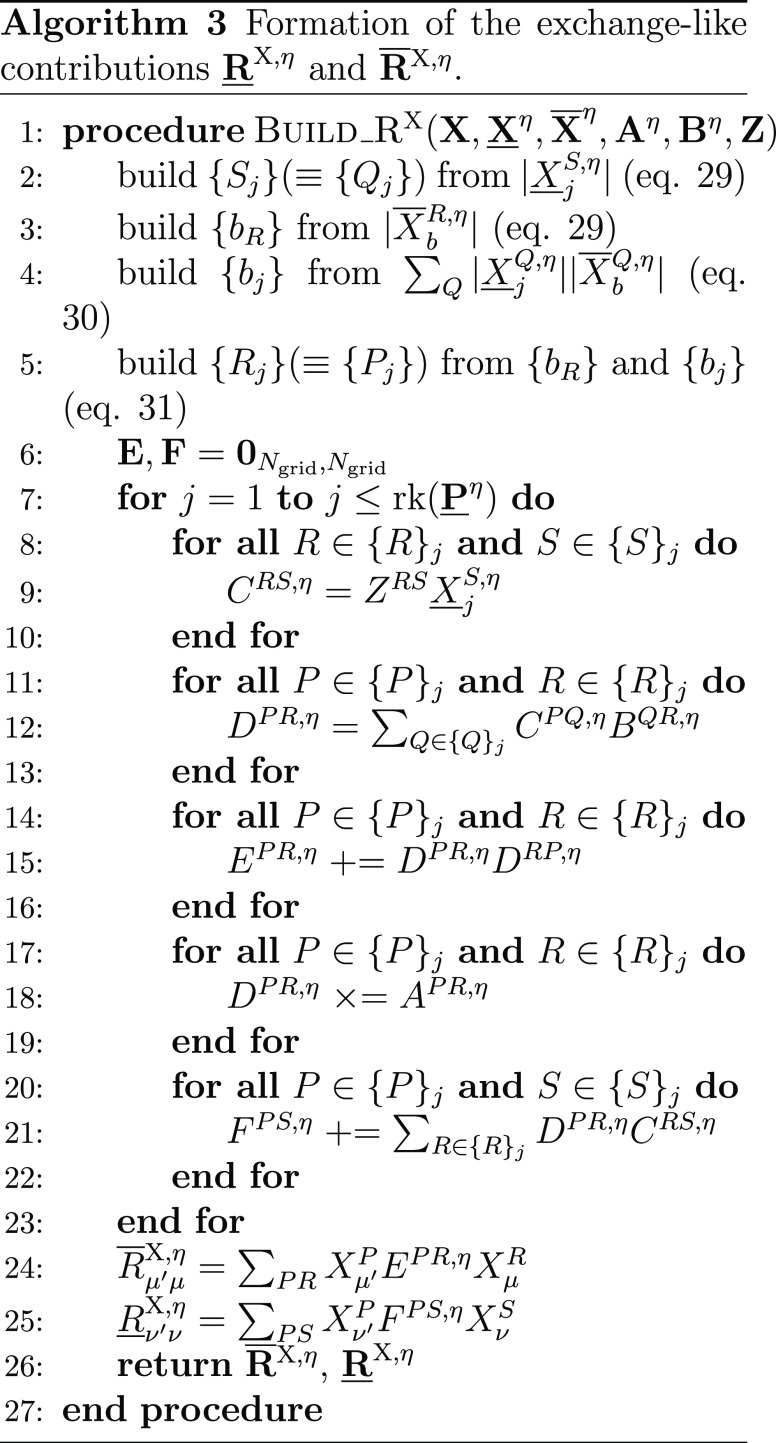


First, according to eqs 29, 30, and 31 all necessary significance lists are computed and then the precursor intermediates to **R̲**^X^ and **R̅**^X^, that is, the matrices **E** and **F**, are accumulated in a loop over the common occupied orbital index *j*. Lines 12 and 21 represent the bottleneck of the algorithm as their evaluation formally scales as . However, since all involved grid indices *P*, *Q*, and *R* are connected to *j* through the significance lists {*S*_*j*_} (identical to {*Q*_*j*_}) and {*R*_*j*_} (identical to {*P*_*j*_}), the size of the involved matrices in NB format is asymptotically constant. Therefore, as *N*_occ_ grows linearly with the molecule size, the formation of the **R**^X^-parts can be done in linear scaling time. For this, appropriate thresholds have to be chosen for the formation of the significance lists. Instead of using the same threshold for all pairs, we identify three different types of lists: (1) grid-occupied orbital index pairs, e.g., {*S*_*j*_}, (2) grid-virtual orbital index pairs, e.g., {*b*_*R*_}, where, in both cases, the orbital index is coupled to the grid index directly by a collocation matrix, and (3) virtual-occupied orbital index pairs, e.g., {*b*_*j*_}, which are coupled in real-space over grid points. While the final algorithm only requires the {*S*_*j*_} and {*R*_*j*_}/{*P*_*j*_} lists, the selection of index pairs included in {*b*_*R*_} and {*b*_*j*_} is still important, as the {*R*_*j*_}/{*P*_*j*_} lists are built from these lists analogously to eq 31.

#### 2.4.3 THC **R**-Matrices: Final Algorithm

Piecing together Algorithms 1 and 2 for the Coulomb-like part and Algorithm 3 for the exchange-like part, the final algorithm for the formation of the **R**-matrices is given by Algorithm 4.
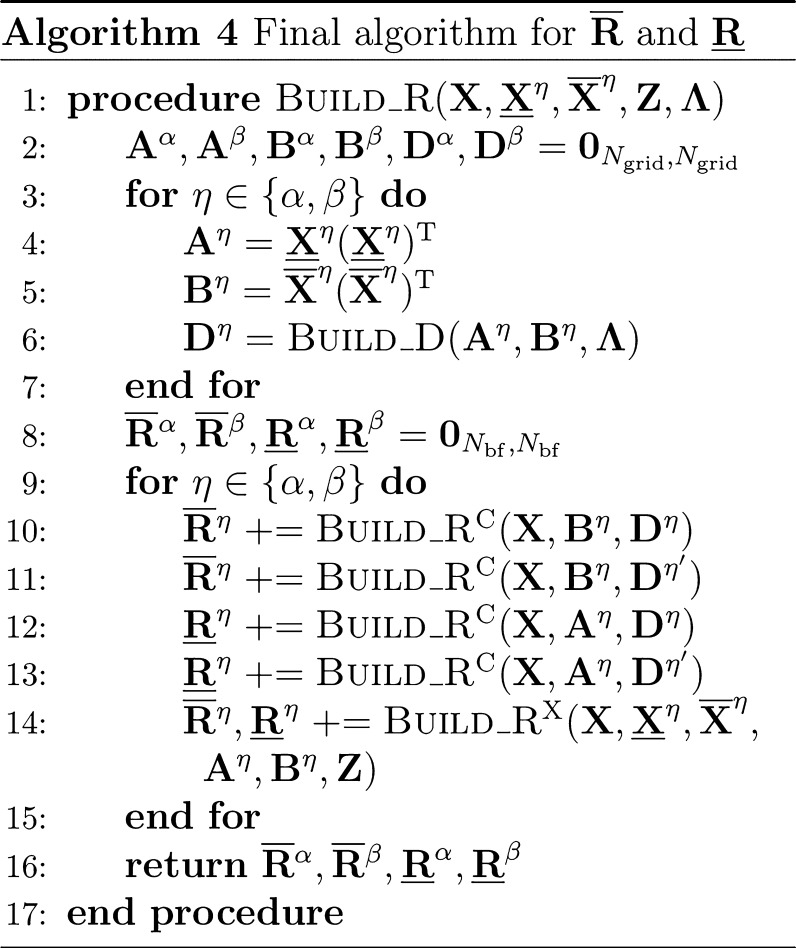


Overall, Algorithm 4 has to be executed once per Laplace point and is separated into a precontraction phase and the phase for the actual formation of the contributions to **R̲** and **R̅**. In the precontraction phase intermediates **D**^η^ are formed, which represent the most expensive part for the Coulomb-like terms, as the subsequent calls to Algorithm 2 only contribute a Schur product and two dgemm operations. These dgemm calls contribute a negligible overhead compared with line 3 of Algorithm 1, as the dimensions of the matrices involved are reduced. In total, however, Algorithm 3 will dominate the runtime for forming the **R**-matrices due to its formal  scaling.

## 3 Computational Details

The above-described THC-ω-RI-CDD-MP2 HFCC code is implemented within our quantum chemistry package FermiONs++.^59−61^ For the THC-based HFCC calculations the hand-optimized grids by Martínez and co-workers^38^ were used together with the Dunning cc-pVXZ (X ∈ {D, T}) basis sets^62^ and the corresponding auxiliary basis sets. For the phosphorus atoms in the DNA backbone, the fluorine grids were used without loss of accuracy as reported in our work on THC-MP2 energies.^41^ All calculations were carried out without the frozen-core approximation. All preceding SCF calculations were converged to an energy difference of 10^–8^ H and a **FPS**−**SPF** commutator difference of 10^–7^ using DIIS acceleration.^63^ For the gradient calculations seven Laplace points were used in the expansion and the DL-UCPSCF algorithm was converged to an error of 10^–4^ for all molecules of the benchmark set in section 4. For all subsequent calculations on larger molecules, a threshold of 10^–3^ was used. These settings were shown to yield errors below 1 MHz.^6,30^ For the assessment of the accuracy of the THC-ω-RI-CDD-MP2 HFCCs against other methods, the standard orientation was used. For the THC factorization of the ERIs an attenuation strength of 0.1 in the attenuated Coulomb metric was used^54,55^ and the same general protocol for screening based on integral partition bounds (IPBs)^64^ and NB, as in our work on THC-MP2 energies, was followed, although adjusted to fit ERIs in the AO basis.^41^ All timings are done on an AMD EPYC 7302 (3.30 GHz) CPU node with 256 GB RAM and 1.7 TB of SSD disk space.

## 4 Results and Discussion

First, the accuracy of the newly developed THC-ω-RI-CDD-MP2 method for the calculation of isotropic HFCCs is assessed against our reference ω-RI-CDD-MP2 implementation.^6,30^ Next, the thresholds necessary for the screening in the expensive exchange-like contribution **R**^X^ are optimized on a set of medium-sized organic radicals. Finally, the scaling of the THC-ω-RI-CDD-MP2 method is analyzed and timings are compared to the ω-RI-CDD-MP2 reference for a set of representative radicals.

### 4.1 Accuracy of THC-ω-RI-CDD-MP2 HFCCs

Throughout this publication, our ω-RI-CDD-MP2 implementation for the computation of HFCCs by Vogler et al.,^6,30^ which was verified against the RI-MP2 implementation in the ORCA program package,^65^ will serve as reference. The original implementation, however, made use of the SOS approximation and excluded the exchange-like terms. To enable a fair comparison, the exchange-like terms were added analogously to the earlier implemented RI-CDD-based AO-MP2 gradient,^6^ albeit with the attenuated Coulomb metric for the RI integrals. For the comparison, first the accuracy of the presented THC-ω-RI-CDD-MP2 method for HFCCs is assessed. The THC-ω-RI-CDD-MP2 method is benchmarked using a set of 12 organic radicals from a recent study^7^ on the effects of electron correlation, molecular dynamic contributions, and solvation effects on HFCCs. Mean absolute deviations (MAD), root-mean-square deviations (RMSD), and absolute maximum deviations (MAX) are given in Table 1. We note that we used all 12 radicals for the comparison, even though, as Vogler et al.^7^ pointed out, some molecules are spin contaminated. While the latter certainly has an effect on the reliability of the results, when comparing to experiment, it should not influence the comparison of different methods.

**Table 1:** Errors of the HFCCs Obtained with the THC-ω-RI-CDD-MP2 Method Compared to the ω-RI-CDD-MP2 Reference Implementation for the HFCC Benchmark Set from Vogler et al.^7^ and the cc-pVXZ/cc-pVXZ-RI (X ∈{D, T}) Basis Sets

basis set	MAD^a^	RMSD^a^	MAX^a^
cc-pVDZ	0.304	0.478	1.822
cc-pVTZ	0.092	0.167	0.675

a Deviations in MHz.

Table 1 shows that the mean errors for the THC-ω-RI-CDD-MP2 method are below 1 MHz for both basis sets, while the MAX error corresponds to atoms with high spin density, that is, ^19^F in the CF_3_ radical for the double-ζ basis set and ^11^B in the BH_3_ radical for the triple-ζ basis set. While for these nuclei the absolute error is larger, the relative error is still below 1%, as due to their high spin density the HFCCs are large in magnitude. The origin of these errors is mainly based on the following two shortcomings: (1) Using the hand-optimized THC grids by Martínez and co-workers^38^ for AO-THC incurs additional errors over MO-THC as these grids were optimized for fitting MO-based ERIs, for which the orbital space is much more compact compared to ERIs in the AO basis. This phenomenon was observed similarly in our recent work on THC-ω-RI-CDD-MP2 energies.^41^ (2) Since grid-based THC uses DFT-like integration grids, THC-based properties are likewise prone to not being rotationally invariant. In DFT the problem is alleviated through larger grids, which THC cannot make use of without forfeiting the reduction in computational cost compared to the respective canonical method. However, we found that these rotational errors are on the order of 0.01−0.05 MHz, depending on the magnitude of the spin density on the respective nucleus. Overall, we consider a mean deviation of less than 1 MHz to be less than the method error of RI-MP2 and certainly accurate enough, when compared against experimental results, where, as shown by Vogler et al.,^7^ other effects like dynamic contributions or solvation effects contribute significantly.

### 4.2 Threshold Optimization

After having established that the presented THC-ω-RI-CDD-MP2 method provides reliable HFCCs, the focus is now on optimizing the time complexity of the underlying algorithm while preserving the accuracy. An obvious point for optimization is the formation of the exchange-like parts **R**^X^, for which Algorithm 3 has quartic scaling if no screening is applied. However, as discussed in section 2.4.2, by applying NB with carefully chosen thresholds, the formation of **R**^X^ should be possible with linear time complexity. As outlined in section 2.4.2, different thresholds will be used in the screening process for the different types of significance lists. We associate the thresholds ε_*Sj*_, ε_*bR*_, and ε_*bj*_ with the significance lists {*S*_*j*_}, {*b*_*R*_}, and {*b*_*j*_}, respectively. Since ε_*Sj*_ directly determines the pairs included in {*S*_*j*_}, and ε_*bR*_/ε_*bj*_ only indirectly determine the pairs in {*R*_*j*_}, the optimization of these thresholds is simplified by separately optimizing ε_*Sj*_ and ε_*bR*_/ε_*bj*_. The thresholds are first optimized for the smaller double-ζ basis set and later transferred to the triple-ζ basis. For this, we chose a set of six medium-sized radical molecules and supramolecular assemblies, which are large enough for the screening to have effect. For further information on this benchmark set see the Supporting Information. Table 2 summarizes the errors and the resulting average number of significant pairs *N̅*_*Sj*_ in {*S*_*j*_} for the optimization of ε_*Sj*_.

**Table 2:** Threshold Optimization of ε_*Sj*_: Errors of the HFCCs and Average Numbers of Significant Pairs (*N̅*_*Sj*_) from the Screening Benchmark Set, Obtained with the Chosen Threshold for ε_*Sj*_ (ε_*bR*_ = 0, ε_*bj*_ = 0, cc-pVDZ)

ε_*Sj*_	*N̅*_*Sj*_^a^	MAD^b^	RMSD^b^	MAX^b^
10^–6^	77.6	2.8 × 10^–7^	8.7 × 10^–7^	1.1 × 10^–5^
10^–5^	57.9	8.2 × 10^–6^	2.0 × 10^–5^	1.8 × 10^–4^
10^–4^	36.7	1.5 × 10^–4^	3.5 × 10^–4^	3.4 × 10^–3^
10^–3^	12.8	3.5 × 10^–3^	1.1 × 10^–2^	1.9 × 10^–1^
10^–2^	5.8	9.2 × 10^–2^	3.6 × 10^–1^	6.2 × 10^0^

a Ratio in %.

b Deviations in MHz.

**Figure 3:**
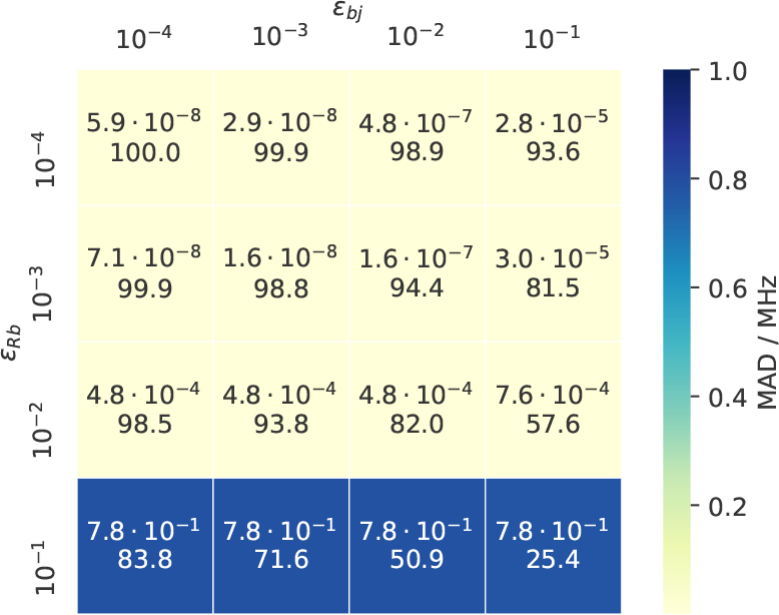
Threshold optimization of ε_*bR*_ and ε_*bj*_: In each cell the MADs (in MHz) of the HFCCs (top value) and the average numbers of significant pairs (*N̅*_*Rj*_ in %, bottom value) are given as an average from the screening benchmark set (ε_*Sj*_ = 0, cc-pVDZ).

While for threshold values of 10^–6^ through 10^–4^ the error remains negligible, the screening shows an effect in that *N̅*_*Sj*_ indicates that only roughly a third of the pairs in {*S*_*j*_} are necessary for this accuracy. The mean errors grow roughly linearly with loosening thresholds and remain sufficiently small for a range of threshold values. For ε_*Sj*_ ≥ 10^–3^ especially the MAX error deteriorates above 1 MHz, while the MAD remains below 0.1 MHz. For the optimization of ε_*bR*_ and ε_*bj*_, combinations of thresholds have to be considered, since they determine, through eq 31, the significant pairs in {*R*_*j*_}. The results of this optimization are shown in Figure 3 as a heatmap.

From Figure 3 it can be seen, that the MAD is stable through a wide range of threshold values, only significantly worsening when choosing ε_*bR*_ > 10^–2^, irrespective of the value chosen for ε_*bj*_. The observation that even for looser thresholds, for example, ε_*bR*_ = ε_*bj*_ = 10^–2^, still roughly 80% of the pairs in {*R*_*j*_} are significant stems from the fact that indices *R* and *j* are coupled indirectly over a virtual orbital *b*. According to eq 31 a pair of indices *R* and *j* is only considered insignificant, if they do not share significant overlap with any virtual orbital *b*. Therefore, *N̅*_*Rj*_ will always be greater than *N̅*_*Sj*_ for any sensibly chosen combination of thresholds.

With the separate optimization of ε_*Sj*_ and ε_*bR*_/ε_*bj*_ as a starting point, different combinations of these three thresholds were tested. The best trade-off between accuracy and the number of significant pairs appeared to be for the thresholds ε_*Sj*_ = 10^–3^, ε_*bR*_ = 10^–2^, and ε_*bj*_ = 10^–2^, for which the errors are summarized in Table 3.

**Table 3:** Errors of the HFCCs from the Screening Benchmark Set, Obtained with the Chosen Thresholds (ε_*Sj*_ = 10^–3^, ε_*bR*_ = 10^–2^, ε_*bj*_ = 10^–2^) for the THC-ω-RI-CDD-MP2 method referenced against the same method with disabled screening

basis set	MAD^a^	RMSD^a^	MAX^a^
cc-pVDZ	0.004	0.011	0.189
cc-pVTZ	0.023	0.077	0.941

a Deviations in MHz.

The chosen combination of thresholds provides good accuracy for both basis sets, with the errors for the triple-ζ basis being somewhat larger. The latter could be improved through a separate optimization of the thresholds for the triple-ζ basis. However, in view of the fact that the mean errors are still below 0.1 MHz, the thresholds optimized for the double-ζ basis set seem to be suitable for the larger basis set as well.

### 4.3 Timings and Scaling

**Figure 4:**
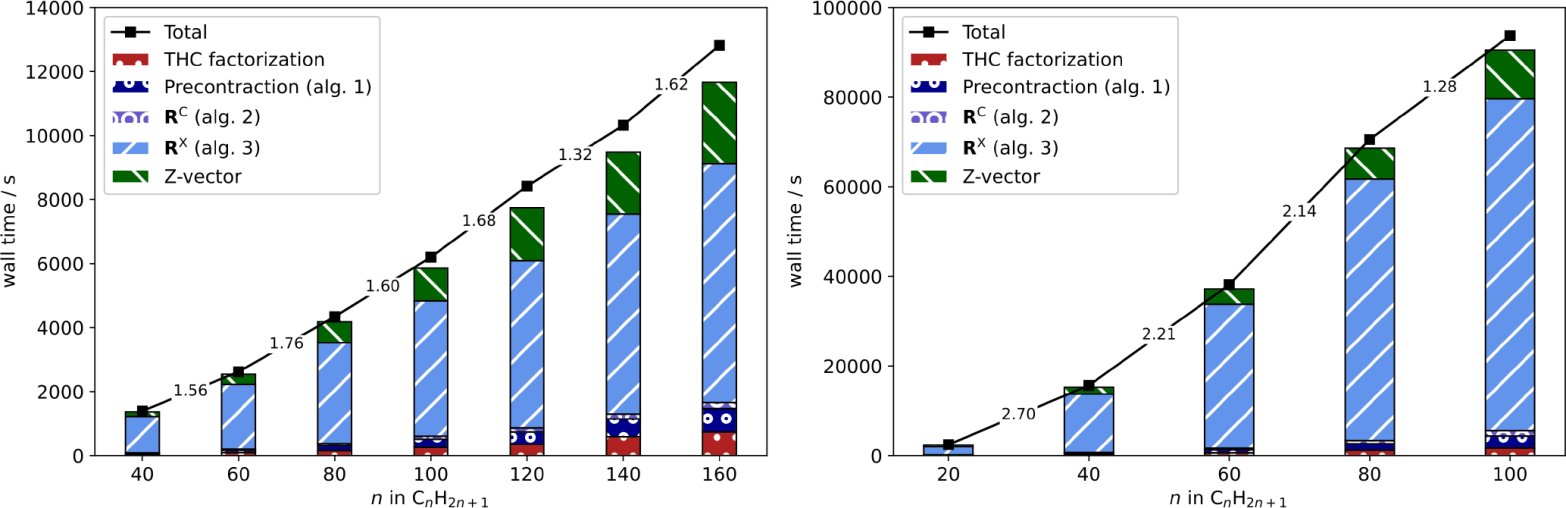
Detailed timings for the computation of HFCCs with the THC-ω-RI-CDD-MP2 method (black) for linear alkyl radicals C_*n*_H_2*n*+1_ as well as significant contributions from underlying steps (colored bars) for the cc-pVDZ (left) and cc-pVTZ (right) basis sets. The numbers between fragments correspond to the scaling with respect to the preceding fragment.

With the optimized screening thresholds at hand, the scaling behavior of the THC-ω-RI-CDD-MP2 method for the computation of HFCCs is analyzed and compared to the previous ω-RI-CDD-MP2 implementation.^6,30^ For the assessment of the asymptotic scaling behavior, HFCCs for a series of linear alkyl radicals C_*n*_H_2*n*+1_ are computed. In Figure 4 the timings are shown together with the underlying contributions from the most significant steps for both basis sets.

As is evident from Figure 4, the overall scaling behavior is governed by the contribution from the exchange-like parts of **R** (Algorithm 3), while the Coulomb-like terms (Algorithms 1 and 2) and the overhead from obtaining the THC factorization only contribute marginally. For both basis sets, the THC-ω-RI-CDD-MP2 method reaches subquadratic scaling, while the scaling is also partly influenced by the Fock matrix builds in the *Z*-vector step. The increased cost of the *Z*-vector step for larger fragment sizes in the case of the double-ζ basis set is also the reason for the overall scaling exponent slightly deteriorating beyond C_140_H_281_ to 1.62. To prevent this unfavorable scaling for the larger triple-ζ basis set, the recommendations by Laqua et al.,^66^ which are default settings in FermiONs++, are followed, and the recently presented seminumerical exchange method (sn-LinK) is used for the exchange part of the Fock matrices. The latter makes the overall scaling for the cc-pVTZ basis set almost entirely be governed by Algorithm 3 (blue bars). Therefore, the scaling reduces to close to linear for the largest fragment size considered. The same is true for the cc-pVDZ basis set, for which Algorithm 3 reaches an apparent asymptotic scaling of 1.3.

To go toward more chemically relevant systems and beyond what was possible with our previous ω-RI-CDD-MP2 implementation, the scaling behavior for spin-labeled adenine–thymine base pair stacks (AT)_*n*_ is assessed.

**Figure 5:**
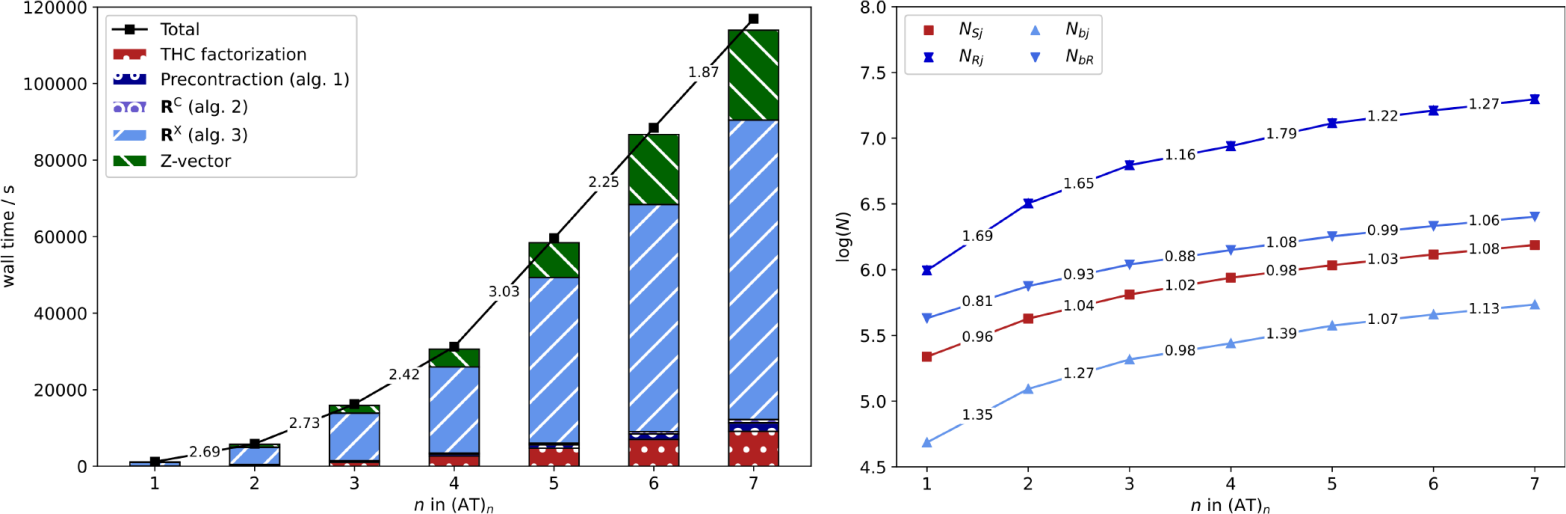
Detailed timings for the computation of HFCCs with the THC-ω-RI-CDD-MP2 method (black) for spin-labeled (AT)_*n*_ radicals as well as significant contributions from underlying steps (colored bars) for the cc-pVDZ basis set. Scaling behavior of the number of significant index pairs {*S*_*j*_} (red) and {*R*_*j*_} (dark blue) for increasing fragment sizes (right). The numbers between fragments correspond to the scaling with respect to the preceding fragment.

Figure 5 (left) shows the scaling behavior for spin-labeled DNA fragments up to seven repetition units or 5101 basis functions. As expected, the onset for subquadratic scaling is for greater fragment sizes compared to the alkyl radicals. Nonetheless, the scaling exponent decreases to 1.87 for (AT)_6_ → (AT)_7_. Further reduction with increasing fragment size can be expected based on the growth rate of the number of significant pairs in {*S*_*j*_} and {*R*_*j*_}. In Figure 5 (right) the logarithm of the total number of significant pairs is shown for all index pairs relevant for Algorithm 3. The runtime of Algorithm 3 is mainly governed by *N*_*Sj*_ and *N*_*Rj*_ and relies on only a constant number of grid points being significant for a given occupied orbital, see section 2.4.2. If only a constant number of grid points is significant for a given occupied orbital, then *N*_*Sj*_ and *N*_*Rj*_ will grow linearly with increasing molecule size. The latter is demonstrated for *N*_*Sj*_, that is, the grid point-occupied orbital pair directly coupled by a collocation matrix. *N*_*Rj*_ is inherently greater than *N*_*Sj*_, since {*R*_*j*_} is formed from eq 31 with coupling of *R* and *j* over a virtual orbital *b*. The latter is also the reason for the scaling exponent not quite reducing to 1.0 and there being an onset for the close to linear scaling. This also explains why Algorithm 3 reaches subquadratic scaling for the DNA system, but not quite linear scaling. Nonetheless, the THC-ω-RI-CDD-MP2 method also reaches subquadratic scaling for the spin-labeled DNA fragments under consideration and allows for the computation of HFCCs for almost 500 atoms and more than 5000 basis functions.

While the asymptotic scaling exponent of a quantum chemical method is certainly important for the treatment of large (bio)chemical systems, the prefactor oftentimes determines the applicability of a method for a certain problem. In other words, a method can be linear scaling, but have a prefactor so large, that calculations still remain unfeasible. Another aspect which determines feasibility are memory/storage requirements, oftentimes governed by the necessity to store ERIs or amplitude tensors present in electron correlation methods. These aspects are considered in the following for a comparison of the ω-RI-CDD-MP2 method in its all-nuclei variant^6,30^ and the presented THC-ω-RI-CDD-MP2 method for a collection of organic radicals. An additional comparison against the selected-nuclei variant of ω-RI-CDD-MP2,^30^ which was proposed to alleviate some of the shortcomings of the ω-RI-CDD-MP2 method, is given in the Supporting Information. Here, the focus is on typical applications on medium-sized organic radicals. Table 4 summarizes the estimated storage requirements *M*_est_ and the wall times *t* for the radicals, as well as the speedups *S* relative to the ω-RI-CDD-MP2 implementation. More information on how the storage requirements are estimated is given in the Supporting Information.

**Table 4:** Comparison of the Memory Requirements *M*_est_, Timings *t*, and Relative Speedups *S* of the THC-ω-RI-CDD-MP2 Method with the Previously Implemented ω-RI-CDD-MP2 Method

		ω-RI-CDD-MP2		THC-ω-RI-CDD-MP2		
system	*N*_bf_	*M*_est_/GB	*t*/h	*M*_est_/GB	*t*/h	*S*
cc-pVDZ						
C_60_H_121_	1445	201.0	101.8	11.7	0.7	145
TEMPO_H_2_O_	1444	208.5	345.9	11.7	1.5	230
(glu)_4_	1074	90.0	380.2	5.9	0.7	540
PTMA_3_	1102	93.8	620.9	6.5	1.2	517
(AT)_2_	1566	286.0	1111.0	23.1	1.9	585
cc-pVTZ						
C_20_H_41_	1174	66.1	363.3	8.7	0.6	606
TEMPO	582	8.2	160.1	2.1	0.2	891
Tyr	530	6.6	99.4	1.4	0.1	780
Thy	1260	86.6	1346.2^a^	8.7	1.1	1224
(AT)_1_	1982	340.4	2727.8^a^	20.7	3.1	880

a Timings are estimated conservatively based on the time taken for the first Laplace point.

THC has a natural advantage over RI-based methods when it comes to storage requirements. In THC-based methods the largest tensors necessary to keep in memory or store on disk are second-order tensors of dimension *N*_grid_ × *N*_grid_. For RI-based methods the dimensionality of the factorized representation of the fourth-order ERI tensor increases to three with dimensions *N*_occ_ × *N*_virt_ × *N*_aux_ in the MO basis. Therefore, THC-ω-RI-CDD-MP2 is superior with an order of magnitude less storage requirements, as can be seen from Table 4. Furthermore, the THC-based method considerably outperforms ω-RI-CDD-MP2 in terms of computation time with speedups up to roughly 600 for the double-ζ basis set and 800–1200 for the triple-ζ basis set. The relative speedups are somewhat reduced when comparing the methods for predominantly linear and very sparse systems like linear alkyl radicals, signifying that the ω-RI-CDD-MP2 uses the sparsity well in these model cases. For larger and more globular structures, the computational savings with the THC-ω-RI-CDD-MP2 method are significantly greater, indicating that Algorithm 4 utilizes the sparsity well, even in nonlinear systems. The latter, and the reduced memory requirements make the THC-ω-RI-CDD-MP2 method attractive for the computation of HFCCs of large, globular systems, as they are commonly encountered in proteins and enzymes. Furthermore, due to the greatly reduced computational cost, the method is applicable in double-hybrid functionals, where usually the MP2 part is the computational bottleneck, while also allowing for sampling of multiple points on the PES.

## 5 Conclusions and Outlook

In this work, we presented the THC-ω-RI-CDD-MP2 method for the efficient and accurate computation of isotropic HFCCs for large organic radicals. As usual for MP2 methods, the exchange-like terms, here **R**^X^, govern the scaling and runtime of the method. This issue was addressed through screening based on the THC collocation matrices **X** in combination with natural blocking for the tensor contractions. An asymptotically linear scaling recipe for the contraction of exchange-like terms in THC format is provided. This recipe is applied to the **R**^X^ terms, reducing the formal quartic scaling to effectively subquadratic, as shown for linear alkyl radicals and spin-labeled DNA strands. The THC-ω-RI-CDD-MP2 method furthermore highlights the attractiveness of THC-based methods for derivatives of electron correlation methods. Derivative calculations usually involve more types of ERIs, for example, half-transformed integrals, integrals with mixed spin in openshell calculations, or integrals contracted with perturbed densities. This generally increases storage requirements compared to single point calculations. Furthermore, the computational cost of the method is strongly increased due to additional ERI contractions. Both challenges are overcome by using THC-factorized ERIs, for which only second-order tensors have to be stored and integral transformations and contractions can be easily reduced to simple dgemm operations. The advantages of THC-based gradient methods are demonstrated for HFCC calculations on a range of medium-sized organic radicals and spin-labeled DNA strands with more than 5000 basis functions.

For future applications, the availability of THC grids has to be improved, as ideally EPR-specific basis sets,^67^ and corresponding THC grids, should be used for the calculations presented. The grids used throughout this publications were hand optimized^38^ and are only viable for the cc-pVXZ (X ∈{D,T}) basis sets. Furthermore, these grids were optimized based on MO-THC-MP2 and not for AO-THC, as used throughout this work. This incurs additional errors due to the larger fitting space. Different techniques for the on-the-fly generation of THC grids have been proposed, either based on PCD of the THC metric in a larger parent grid basis,^40^ or based on centroidal Voronoi tesselation (CVT),^36^ and could be applied for the calculation of THC-MP2 HFCCs in future work.

Finally, the developed THC-ω-RI-CDD-MP2 method can easily be used for the MP2 part of double-hybrid functionals and, once appropriate grids are available, enable accurate HFCC predictions for large molecules. Furthermore, due to the reduced computational complexity and memory requirements, THC-ω-RI-CDD-MP2, in conjunction with an appropriate double-hybrid functional, is an attractive candidate for QM/MM HFCC calculations.

## Supporting Information Available

Detailed algorithm for the asymptotically linear scaling formation of THC-MP2 exchange-like energy contribution as well as the individual formation of **R̅**^X^ and **R̲**^X^, detailed results for the benchmark calculations, details on the memory requirement estimation and comparison of selected-nuclei ω-RI-CDD-MP2 against THC-ω-RI-CDD-MP2 (PDF)
Structures (ZIP)
