# Supplementary material for: Tensor-Hypercontracted MP2 First Derivatives: Runtime and Memory Efficient Computation of Hyperfine Coupling Constants
Source: J Chem Theory Comput. 2022 Aug 9;18(9):5233–45. doi: 10.1021/acs.jctc.2c00118 (PMC9476664; doi:10.1021/acs.jctc.2c00118)
Supplement: Supplementary file 1 — ct2c00118_si_001.pdf [file ct2c00118_si_001.pdf]

Supporting Information: Tensor-hypercontracted MP2 First Derivatives: Runtime and Memory Efficient Computation of Hyperfine Coupling Constants

Felix H. Bangerter,[†] Michael Glasbrenner,[†] and Christian Ochsenfeld[*,†,‡]

†*Chair of Theoretical Chemistry, Department of Chemistry, University of Munich (LMU), D-81377 Munich, Germany*

‡*Max Planck Institute for Solid State Research, D-70569 Stuttgart, Germany*

E-mail: christian.ochsenfeld@cup.uni-muenchen.de

Contents

1 Alternative Derivation of the Analytical Expression for the THC Z-Matrix S-3

2 THC-MP2 Exchange-like Contractions S-4

3 Exchange Contribution to $\overline{R}_{\mu'\mu}$ S-5

4 Exchange Contribution to $\underline{R}_{\nu'\nu}$ S-6

5 Detailed Results for the Accuracy Benchmark S-8

6 Estimation of Memory Requirements S-12

7 Comparison of (Selected-nuclei) ω-RI-CDD-MP2 and THC-ω-RI-CDD-MP2 S-14

7.1 Asymptotic Scaling Comparison S-14

7.2 Timing Comparison With Selected-nuclei ω-RI-CDD-MP2 . . . . S-15

8 Structures S-17

8.1 Benchmark Set of Small Organic Radicals S-17

8.2 Benchmark Set of Medium-sized Organic Radicals S-17

8.3 Test Set for the Comparison Against ω-RI-CDD-MP2 . . . . S-17

References S-19

1 Alternative Derivation of the Analytical Expression for the THC Z-Matrix

The AO-THC least-squares objective function[S1] is given by

$$O = \frac{1}{2}\left\|(\mu\nu|\lambda\sigma) - \sum_{PQ} X_\mu^P X_\nu^P Z^{PQ} X_\lambda^Q X_\sigma^Q\right\|_2^2, \tag{1}$$

which is quartic in the collocation matrix $\mathbf{X}$ and linear in the grid representation of the $1/r$ operator, i.e., $\mathbf{Z}$. The LS problem can be generalized to

$$O = \frac{1}{2}\left\|\mathbf{B} - \mathbf{A}\mathbf{Y}\mathbf{A}^{\mathrm{T}}\right\|_2^2, \tag{2}$$

where $\mathbf{B} \in \mathbb{R}^{n\times n}$, $\mathbf{A} \in \mathbb{R}^{n\times m}$ and $\mathbf{Y} \in \mathbb{R}^{m\times m}$, with $n = N_{\mathrm{bf}}^2$ and $m = N_{\mathrm{grid}}$ in the case of LS-THC. The analytic expression for the THC $\mathbf{Z}$ matrix given by Martínez and coworkers[S1] is then simply the solution of the normal equations associated with eq 1. In general notation, the normal equations associated with the LS problem of eq 2 are simply given as

$$\mathbf{A}^{\mathrm{T}}\mathbf{A}\mathbf{Y}\mathbf{A}^{\mathrm{T}}\mathbf{A} = \mathbf{A}^{\mathrm{T}}\mathbf{B}\mathbf{A}. \tag{3}$$

To solve for $\mathbf{Y}$, the moment matrix $\mathbf{A}^{\mathrm{T}}\mathbf{A}$ has to be inverted and the solution is given by

$$\mathbf{Y} = (\mathbf{A}^{\mathrm{T}}\mathbf{A})^{-1}\mathbf{A}^{\mathrm{T}}\mathbf{B}\mathbf{A}(\mathbf{A}^{\mathrm{T}}\mathbf{A})^{-1}. \tag{4}$$

If the substitutions $\mathbf{A} = \mathbf{R}$, where $R_{\mu\nu}^P \equiv X_\mu^P X_\nu^P$, $\mathbf{B} = \boldsymbol{\mathcal{I}}$ and $\mathbf{Y} = \mathbf{Z}$ are made, the familiar analytic expression for the THC $\mathbf{Z}$-tensor is obtained

$$\begin{aligned} \mathbf{Z} &= (\mathbf{R}^{\mathrm{T}}\mathbf{R})^{-1}\mathbf{R}^{\mathrm{T}}\boldsymbol{\mathcal{I}}\mathbf{R}(\mathbf{R}^{\mathrm{T}}\mathbf{R})^{-1} \\ &= \mathbf{S}^{-1}\mathbf{E}\mathbf{S}^{-1}, \end{aligned} \tag{5}$$

where $\mathbf{S} \equiv \mathbf{R}^{\mathrm{T}}\mathbf{R}$ is the THC grid metric, $\boldsymbol{\mathcal{I}}$ is the matrix representation of the ERI tensor

and $\mathbf{E} \equiv \mathbf{R}^{\mathrm{T}}\boldsymbol{\mathcal{I}}\mathbf{R}$ is the grid-projected ERI tensor.

2 THC-MP2 Exchange-like Contractions

The proposed algorithm for the computation of the exchange-like energy contribution for the THC-ω-RI-CDD-MP2 method follows the same ideas as the algorithm for the exchange-like contribution $\mathbf{R}^{\mathrm{X}}$ from the main part of this publication. Note, that this algorithm is applicable to all types of methods derived from THC-CDD-MP2, as the RI approximation is only used during the formation of the THC factorization.

Algorithm 1 Compute the exchange-like MP2 energy $E_{\text{MP2-K}}^{\text{THC-CDD}}$

procedure COMPUTE_THC_MP2_X($\mathbf{X}, \mathbf{Z}$)
    $E_{\text{MP2-K}} = 0.0$
    for $\kappa = 1$ **to** N_κ **do** ▷ Laplace quadrature
        $\underline{L}_{\mu i}, \overline{L}_{\nu a} =$ GET_CHOLESKY_FACTORS(κ)
        $\underline{X}_i^P = \sum_\mu \underline{L}_{\mu i} X_\mu^P$
        $\overline{X}_a^P = \sum_\nu \overline{L}_{\nu a} X_\nu^P$
        $A^{PR} = \sum_i \underline{X}_i^P \underline{X}_i^R$
        $B^{PS} = \sum_a \overline{X}_a^P \overline{X}_a^S$
        build $\{S_j\}$ from $|\underline{X}_j^S|$
        build $\{a_P\}$ from $|\overline{X}_a^P|$
        build $\{a_j\}$ from $\sum_S |\underline{X}_j^S||\overline{X}_a^S|$
        build $\{P_j\}$ from $\{a_P\}$ and $\{a_j\}$ ▷ identical to $\{R_j\}$
        $e_{\text{MP2-K}} = 0.0$
        for $j = 1$ **to** $j \leq \text{rk}(\underline{\mathbf{P}})$ **do** ▷ batching over occupied index j
            for all $P \in \{P\}_j$ **and** $S \in \{S\}_j$ **do**
                $C^{PS} = B^{PS}\underline{X}_j^S$
            end for
            for all $P \in \{P\}_j$ **and** $R \in \{R\}_j$ **do**
                $D^{PR} = \sum_{S \in \{S\}_j} Z^{RS} C^{PS}$
            end for
            $e_{\text{MP2-K}} \mathrel{+}= \sum_{P \in \{P\}_j} \sum_{R \in \{R\}_j} A^{PR} D^{PR} D^{RP}$
        end for
        $E_{\text{MP2-K}} \mathrel{+}= e_{\text{MP2-K}}$
    end for
    return $E_{\text{MP2-K}}$
end procedure

Asymptotic linear scaling of Algorithm 1 is exemplarily demonstrated for linear alkane chains

up to $C_{120}H_{242}$ in Figure S1.

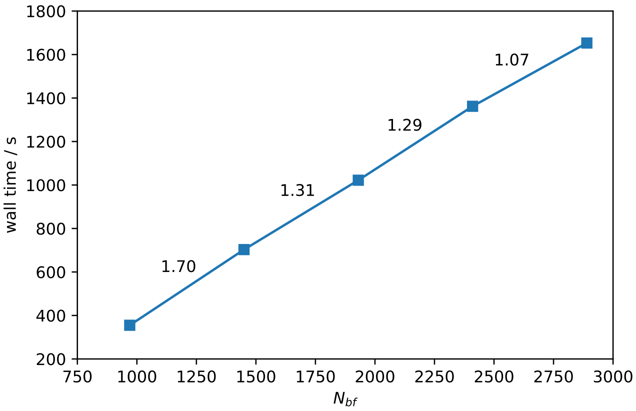

Figure S1: Wall times of Algorithm 1 for linear alkanes C_nH_{2n+2} (n ∈ {40, 60, 80, 100, 120}) and the cc-pVDZ basis set. Black numbers between fragments correspond to the scaling with respect to the preceding fragment.

3 Exchange Contribution to $\overline{R}_{\mu'\mu}$

The exchange-like contribution to $\overline{\mathbf{R}}$ for a single Laplace point is given by

$$\begin{aligned}\overline{R}^{\mathrm{X},\eta}_{\mu'\mu} &= \sum_j \sum_{ab} (\mu'\overline{a}|\underline{j}\overline{b})_{\eta\eta} (\mu\overline{b}|\underline{j}\overline{a})_{\eta\eta} \\ &\approx \sum_j \sum_{ab} \sum_{PQRS} X^P_{\mu'} \overline{X}^{P,\eta}_a Z^{PQ} \underline{X}^{Q,\eta}_j \overline{X}^{Q,\eta}_b \cdot X^R_{\mu} \overline{X}^{R,\eta}_b Z^{RS} \underline{X}^{S,\eta}_j \overline{X}^{S,\eta}_a,\end{aligned} \tag{6}$$

and can be efficiently obtained by the following algorithm:

Algorithm 2 Compute the exchange-like contribution to $\overline{R}_{\mu'\mu}$

procedure BUILD_RMUMUX($\mathbf{X}, \mathbf{Z}, \underline{\mathbf{X}}, \overline{\mathbf{X}}$)
  build $\{S_j\}$ from $|\underline{X}_j^{S,\eta}|$
  build $\{a_P\}$ from $|\overline{X}_a^{P,\eta}|$
  build $\{a_j\}$ from $\sum_S |\underline{X}_j^{S,\eta}||\overline{X}_a^{S,\eta}|$
  build $\{P_j\}$ from $\{a_P\}$ and $\{a_j\}$ ▷ identical to $\{R_j\}$
  $B^{PS,\eta} = \sum_a \overline{X}_a^{P,\eta} \overline{X}_a^{S,\eta}$
  $E^{PR,\eta} = \mathbf{0}_{N_\mathrm{grid}, N_\mathrm{grid}}$
  for $j = 1$ **to** $j \leq \mathrm{rk}(\underline{\mathbf{P}}^\eta)$ **do** ▷ batching over occupied index j
    for all $R \in \{R\}_j$ **and** $S \in \{S\}_j$ **do**
      $C^{RS,\eta} = Z^{RS} \underline{X}_j^{S,\eta}$
    end for
    for all $P \in \{P\}_j$ **and** $R \in \{R\}_j$ **do**
      $D^{PR,\eta} = \sum_{S \in \{S\}_j} C^{RS,\eta} B^{PS,\eta}$
    end for
    for all $P \in \{P\}_j$ **and** $R \in \{R\}_j$ **do**
      $E^{PR,\eta}$ += $D^{PR,\eta} D^{RP,\eta}$
    end for
  end for
  $\overline{R}_{\mu'\mu}^{\mathrm{X},\eta} = \sum_{PR} X_{\mu'}^P E^{PR,\eta} X_\mu^R$
  return $\overline{R}_{\mu'\mu}^{\mathrm{X},\eta}$
end procedure

4 Exchange Contribution to $\underline{R}_{\nu'\nu}$

The exchange-like contribution to $\underline{\mathbf{R}}$ for a single Laplace point is given by

$$\begin{aligned} \underline{R}_{\nu'\nu}^{\mathrm{X},\eta} &= \sum_{ij} \sum_{b} (\underline{i}\nu'|\underline{j}\overline{b})_{\eta\eta} (\underline{i}\overline{b}|\underline{j}\nu)_{\eta\eta} \\ &\approx \sum_{ij} \sum_{b} \sum_{PQRS} \underline{X}_i^{P,\eta} X_{\nu'}^P Z^{PQ} \underline{X}_j^{Q,\eta} \overline{X}_b^{Q,\eta} \cdot \underline{X}_i^{R,\eta} \overline{X}_b^{R,\eta} Z^{RS} \underline{X}_j^{S,\eta} X_\nu^S . \end{aligned} \tag{7}$$

By symmetry, the exchange contribution $\underline{R}_{\nu'\nu}^{\mathrm{X},\eta}$, given by equation 7, can be computed analogously to the exchange contribution $\overline{R}_{\mu'\mu}^{\mathrm{X},\eta}$, where the batching loop iterates over all virtual MOs b instead of all occupied MOs j. This, however, will increase the prefactor significantly as $N_\mathrm{virt} \approx N_\mathrm{bf} \gg N_\mathrm{occ}$. If instead the batching loop is kept over all occupied orbitals j, an additional Schur product and an additional matrix multiplication have to be performed,

compared to the algorithm for $\overline{R}^{\mathrm{X},\eta}_{\mu'\mu}$. The number of floating point operations (FLOPs) for the batching over b is roughly $2N_{\mathrm{virt}}N^2_{\mathrm{grid}} + N_{\mathrm{virt}}N^3_{\mathrm{grid}}$ (the scaling of $\mathbf{Z}$, a matrix multiplication and the accumulation in $\mathbf{E}$ for every b), whereas for the batching over j the FLOP count is $2N_{\mathrm{occ}}N^2_{\mathrm{grid}} + 2N_{\mathrm{occ}}N^3_{\mathrm{grid}}$ (see Algorithm 3). It can easily be shown that the FLOP ratio is roughly $\frac{2N_{\mathrm{occ}}}{N_{\mathrm{virt}}}$ in favor of the batching over j. Therefore, the exchange-like contribution to $\overline{\mathbf{R}}$ for a single Laplace point can efficiently be computed with the following algorithm:

Algorithm 3 Compute the exchange-like contribution to $\underline{R}_{\nu'\nu}$

procedure BUILD_RNUNUX($\mathbf{X}, \mathbf{Z}, \underline{\mathbf{X}}, \overline{\mathbf{X}}$)
  build $\{S_j\}$ from $|\underline{X}^{S,\eta}_j|$
  build $\{b_R\}$ from $|\overline{X}^{R,\eta}_b|$
  build $\{b_j\}$ from $\sum_Q |\underline{X}^{Q,\eta}_j||\overline{X}^{Q,\eta}_b|$
  build $\{R_j\}$ from $\{b_R\}$ and $\{b_j\}$ ▷ identical to $\{P_j\}$
  $A^{PR,\eta} = \sum_i \underline{X}^{P,\eta}_i \underline{X}^{R,\eta}_i$
  $B^{QR,\eta} = \sum_b \overline{X}^{Q,\eta}_b \overline{X}^{R,\eta}_b$
  $E^{PS,\eta} = \mathbf{0}_{N_{\mathrm{grid}},N_{\mathrm{grid}}}$
  for $j = 1$ **to** $j \leq \mathrm{rk}(\underline{\mathbf{P}}^{\eta})$ **do** ▷ batching over occupied index j
    for all $R \in \{R\}_j$ **and** $S \in \{S\}_j$ **do**
      $C^{RS,\eta} = Z^{RS}\underline{X}^{S,\eta}_j$ ▷ identical to C^{PQ}
    end for
    for all $P \in \{P\}_j$ **and** $R \in \{R\}_j$ **do**
      $D^{PR,\eta} = \sum_{Q\in\{Q\}_j} C^{PQ,\eta}B^{QR,\eta}$
    end for
    for all $P \in \{P\}_j$ **and** $R \in \{R\}_j$ **do**
      $D^{PR,\eta} \mathrel{\times}= A^{PR,\eta}$
    end for
    for all $P \in \{P\}_j$ **and** $S \in \{S\}_j$ **do**
      $E^{PS,\eta} \mathrel{+}= \sum_{R\in\{R\}_j} D^{PR,\eta}C^{RS,\eta}$
    end for
  end for
  $\underline{R}^{\mathrm{X},\eta}_{\nu'\nu} = \sum_{PS} X^P_{\nu'}E^{PS,\eta}X^S_{\nu}$
  return $\underline{R}^{\mathrm{X},\eta}_{\nu'\nu}$
end procedure

Finally, by closer inspection of algorithms 2 and 3 it can be seen that they share common intermediates, which makes a joined computation of $\overline{R}^{\mathrm{X},\eta}_{\mu'\mu}$ and $\underline{R}^{\mathrm{X},\eta}_{\nu'\nu}$ attractive, as explained in the main part of this publication.

5 Detailed Results for the Accuracy Benchmark

Table S1: Detailed comparison of THC-ω-RI-CDD-MP2 HFCCs against ω-RI-CDD-MP2 for the benchmark set by Vogler *et al.*[S2] and the cc-pVDZ/cc-pVDZ-RI basis set combination.

		ω-RI-CDD-MP2	THC-ω-RI-CDD-MP2		
radical	nucleus	A_{iso} / MHz	A_{iso} / MHz	ΔA_{iso} / MHz	ΔA_{iso} / %
**1**	^{14}N	67.454	67.422	0.032	0.05
	^{13}C	-36.580	-36.601	0.021	0.06
	^{1}H^{a}	94.085	94.150	0.065	0.07
	^{1}H^{b}	-3.286	-3.316	0.030	0.91
**2**	^{14}N	66.966	67.269	0.303	0.45
	^{13}C ($\underline{C}$H)	-28.521	-28.999	0.478	1.68
	^{13}C^{a} ($\underline{C}H_3$)	59.983	60.232	0.249	0.42
	^{13}C^{b} ($\underline{C}H_3$)	10.989	11.186	0.197	1.79
	^{1}H (C$\underline{H}$)	25.150	25.414	0.264	1.05
	^{1}H^{a} ($C\underline{H}_3$)	-0.186	-0.206	0.020	10.75
	^{1}H^{b} ($C\underline{H}_3$)	3.194	3.127	0.067	2.10
**3**	^{13}C	-17.038	-17.152	0.114	0.67
	^{1}H^{a}	346.221	346.092	0.129	0.04
	^{1}H^{b}	-19.814	-19.807	0.007	0.04
**4**	^{13}C ($\underline{C}H_3$)	-11.972	-11.991	0.019	0.16
	^{13}C ($\underline{C}H_2$)	43.314	43.340	0.026	0.06
	^{1}H ($C\underline{H}_2$)	-2.180	-2.141	0.039	1.79
	^{1}H^{a} ($C\underline{H}_3$)	12.536	12.557	0.021	0.17
	^{1}H^{b} ($C\underline{H}_3$)	150.898	151.181	0.283	0.19
**5**	^{14}N	-5.277	-4.714	0.563	10.67
	o-^{13}C	59.599	59.003	0.596	1.00
	m-^{13}C	2.734	2.951	0.217	7.94
	p-^{13}C	27.022	26.429	0.593	2.19
	o-^{1}H	-34.018	-33.485	0.533	1.57
	m-^{1}H	-5.813	-6.188	0.375	6.45
	p-^{1}H	-26.106	-25.747	0.359	1.38
**6**	^{14}N	145.403	146.474	1.071	0.74
	^{17}O	-78.435	-78.963	0.528	0.67

**7**	^{13}C	160.828	160.825	0.003	0.00
	^{1}H	-72.879	-72.873	0.006	0.01
**8**	^{17}O	-121.219	-120.697	0.522	0.43
	^{13}C (H$\underline{\mathrm{C}}$O)	-92.061	-92.184	0.123	0.13
	^{13}C ($\underline{\mathrm{C}}\mathrm{H}_3$)	55.048	55.202	0.154	0.28
	^{1}H ($\underline{\mathrm{H}}$CO)	296.678	297.826	1.148	0.39
	^{1}H ($\mathrm{C}\underline{\mathrm{H}}_3$)	-7.866	-7.870	0.004	0.05
**9**	^{13}C	665.612	666.734	1.122	0.17
	^{19}F	432.589	434.411	1.822	0.42
**10**	^{13}C ($\underline{\mathrm{C}}$H)	300.933	300.431	0.502	0.17
	^{13}C ($\underline{\mathrm{C}}\mathrm{H}_2$)	25.532	26.189	0.657	2.57
	^{1}H (C$\underline{\mathrm{H}}$)	57.738	57.880	0.142	0.25
	^{1}H (Z-$\mathrm{C}\underline{\mathrm{H}}_2$)	109.551	109.340	0.211	0.19
	^{1}H (E-$\mathrm{C}\underline{\mathrm{H}}_2$)	57.501	57.349	0.152	0.26
**11**	^{13}C	-11.389	-11.089	0.300	2.63
	^{14}N	35.343	35.348	0.005	0.01
	^{1}H	158.759	158.818	0.059	0.04
**12**	^{11}B	352.047	352.144	0.097	0.03
	^{1}H	21.757	21.694	0.063	0.29

Table S2: Detailed comparison of THC-ω-RI-CDD-MP2 HFCCs against ω-RI-CDD-MP2 for the benchmark set by Vogler *et al.*[S2] and the cc-pVTZ/cc-pVTZ-RI basis set combination.

		ω-RI-CDD-MP2	THC-ω-RI-CDD-MP2		
radical	nucleus	A_{iso} / MHz	A_{iso} / MHz	ΔA_{iso} / MHz	ΔA_{iso} / %
**1**	^{14}N	19.036	18.984	0.052	0.27
	^{13}C	-32.174	-32.145	0.029	0.09
	^{1}H^{a}	98.541	98.156	0.385	0.39
	^{1}H^{b}	-3.236	-3.227	0.009	0.28
**2**	^{14}N	19.046	19.028	0.018	0.09
	^{13}C ($\underline{C}$H)	-24.324	-24.196	0.128	0.53
	^{13}C^{a} ($\underline{C}H_3$)	64.018	64.008	0.010	0.02
	^{13}C^{b} ($\underline{C}H_3$)	10.865	10.871	0.006	0.06
	^{1}H (C$\underline{H}$)	25.437	25.432	0.005	0.02
	^{1}H^{a} ($C\underline{H}_3$)	0.085	0.085	0.000	0.00
	^{1}H^{b} ($C\underline{H}_3$)	3.632	3.633	0.001	0.03
**3**	^{13}C	-44.461	-44.477	0.016	0.04
	^{1}H^{a}	374.836	374.851	0.015	0.00
	^{1}H^{b}	-19.968	-19.966	0.002	0.01
**4**	^{13}C ($\underline{C}H_3$)	-14.272	-14.278	0.006	0.04
	^{13}C ($\underline{C}H_2$)	-15.463	-15.498	0.035	0.23
	^{1}H ($C\underline{H}_2$)	0.306	0.323	0.017	5.56
	^{1}H^{a} ($C\underline{H}_3$)	14.002	14.001	0.001	0.01
	^{1}H^{b} ($C\underline{H}_3$)	162.255	162.255	0.000	0.00
**5**	^{14}N	4.593	4.485	0.108	2.35
	o-^{13}C	1.599	1.857	0.258	16.14
	m-^{13}C	-28.914	-29.369	0.545	1.88
	p-^{13}C	19.337	19.614	0.277	1.43
	o-^{1}H	-30.602	-30.675	0.073	0.24
	m-^{1}H	-3.842	-3.720	0.122	3.18
	p-^{1}H	-27.272	-27.391	0.119	0.44
**6**	^{14}N	129.520	129.454	0.066	0.05
	^{17}O	-53.697	-53.767	0.070	0.13
**7**	^{13}C	22.741	22.705	0.036	0.16

	^{1}H	-68.648	-68.651	0.003	0.00
**8**	^{17}O	-7.694	-8.519	0.175	2.27
	^{13}C (H$\underline{C}$O)	-86.133	-86.369	0.236	0.27
	^{13}C ($\underline{C}H_3$)	48.003	48.027	0.024	0.05
	^{1}H ($\underline{H}$CO)	311.223	311.200	0.023	0.01
	^{1}H (C$\underline{H}_3$)	-7.700	-7.746	0.046	0.60
**9**	^{13}C	666.302	666.191	0.111	0.02
	^{19}F	373.470	374.320	0.150	0.04
**10**	^{13}C ($\underline{C}$H)	211.780	211.585	0.195	0.09
	^{13}C ($\underline{C}H_2$)	49.607	49.648	0.041	0.08
	^{1}H (C$\underline{H}$)	57.362	57.365	0.003	0.01
	^{1}H (Z-C$\underline{H}_2$)	115.421	115.412	0.009	0.01
	^{1}H (E-C$\underline{H}_2$)	71.645	71.667	0.022	0.03
**11**	^{13}C	-8.745	-8.760	0.015	0.17
	^{14}N	-10.393	-10.522	0.129	1.24
	^{1}H	158.701	158.740	0.039	0.02
**12**	^{11}B	326.791	327.116	0.675	0.21
	^{1}H	23.902	23.886	0.016	0.07

Notes:

- the enumeration of radicals is identical to the enumeration used by Vogler *et al.*[S2]
- radicals **2** and **8**: the HFCCs for the ^{1}H nuclei belonging to the same methyl-group were averaged
- radicals **3** and **4**: due to symmetry there are a set of 4 and a set of 2 (close to) identical ^{1}H nuclei across both methyl-groups

For further notes on symmetry breaking due to Jahn-Teller effects in these molecules refer to the work by Vogler *et al.*[S2]

6 Estimation of Memory Requirements

When estimating the memory requirements for the ω-RI-CDD-MP2 method and its selected-nuclei variant by Vogler *et al.*,[S3] and the THC-ω-RI-CDD-MP2 method from the main part of this publication, only the highest-order tensors are considered. Without taking the integral screening into account, the memory footprint of the ω-RI-CDD-MP2 is governed by the **B**-intermediates, exemplarily given by

$$B^{\eta}_{\underline{i}\overline{a},\beta} = \sum_{\alpha} (\underline{i}\overline{a}|\alpha)_{\eta} (\alpha|\beta)^{-1/2}, \tag{8}$$

for fully MO-transformed three-center RI integrals. Besides the fully transformed $B_{\underline{i}\overline{a},\beta}$-intermediate and its permutation $B_{\overline{a}\underline{i},\beta}$ (needed for exchange-type contributions), also the half-transformed intermediates $B_{\mu\overline{a},\beta}$ and $B_{\underline{i}\nu,\beta}$ are needed. All four **B**-type intermediates are needed for the α and β electrons, amounting to a total of eight third-order tensors. Therefore, the memory requirements in GB, assuming double precision floating point arithmetic, can be estimated as

$$\begin{aligned} M_{\mathrm{est},1} &= \underbrace{2}_{\alpha/\beta} \times \left(2 \times \underbrace{N_{\mathrm{occ}} \times N_{\mathrm{virt}} \times N_{\mathrm{aux}}}_{B_{\underline{i}\overline{a},\beta}/B_{\overline{a}\underline{i},\beta}} + \underbrace{N_{\mathrm{bf}} \times N_{\mathrm{virt}} \times N_{\mathrm{aux}}}_{B_{\mu\overline{a},\beta}} + \underbrace{N_{\mathrm{occ}} \times N_{\mathrm{bf}} \times N_{\mathrm{aux}}}_{B_{\underline{i}\nu,\beta}} \right) \times \underbrace{\frac{8\,\mathrm{B}}{1024^3\,\frac{\mathrm{B}}{\mathrm{GB}}}}_{\rightarrow \mathrm{GB}} \\ &= 2\,N_{\mathrm{aux}}\,(2N_{\mathrm{occ}}N_{\mathrm{virt}} + N_{\mathrm{bf}}N_{\mathrm{virt}} + N_{\mathrm{occ}}N_{\mathrm{bf}})\,\frac{8\,\mathrm{B}}{1024^3\,\frac{\mathrm{B}}{\mathrm{GB}}} \\ &= 2\,N_{\mathrm{aux}}\left(2N_{\mathrm{occ}}N_{\mathrm{virt}} + N_{\mathrm{bf}}^2\right)\frac{8\,\mathrm{B}}{1024^3\,\frac{\mathrm{B}}{\mathrm{GB}}}. \end{aligned} \tag{9}$$

The memory requirements for the selected-nuclei variant are similar, except that here additional **B**-intermediates half-transformed with the perturbed density matrix are required, which results in

$$
\begin{aligned}
M_{\text{est},2} = &\underbrace{2}_{\alpha/\beta} \times \left(2 \times \underbrace{N_{\text{occ}} \times N_{\text{virt}} \times N_{\text{aux}}}_{B_{\underline{i}\overline{a},\beta}/B_{\overline{a}\underline{i},\beta}} + 2 \times \underbrace{N_{\text{bf}} \times N_{\text{virt}} \times N_{\text{aux}}}_{B_{\mu\overline{a},\beta}/B_{\mu^x\overline{a},\beta}} + 2 \times \underbrace{N_{\text{occ}} \times N_{\text{bf}} \times N_{\text{aux}}}_{B_{\underline{i}\nu,\beta}/B_{\underline{i}\nu^x,\beta}} \right) \\
&\times \underbrace{\frac{8\,\text{B}}{1024^3\,\frac{\text{B}}{\text{GB}}}}_{\to\text{GB}} \\
&= 4\,N_{\text{aux}} \left(N_{\text{occ}}N_{\text{virt}} + N_{\text{bf}}N_{\text{virt}} + N_{\text{occ}}N_{\text{bf}} \right) \frac{8\,\text{B}}{1024^3\,\frac{\text{B}}{\text{GB}}} \\
&= 4\,N_{\text{aux}} \left(N_{\text{occ}}N_{\text{virt}} + N_{\text{bf}}^2 \right) \frac{8\,\text{B}}{1024^3\,\frac{\text{B}}{\text{GB}}}.
\end{aligned}
\tag{10}
$$

For the THC-ω-RI-CDD-MP2 method, assuming the three-center integrals are not kept in memory after obtaining the THC factorized ERIs, the most memory demanding intermediates are second-order tensors of dimension $N_{\text{grid}} \times N_{\text{grid}}$. Since the algorithm for the exchange-like contribution to the $\mathbf{R}$-matrices requires more such intermediates and since the memory occupied by the algorithm for the Coulomb-like contribution can be reclaimed, the memory requirements are estimated based on the algorithm for the exchange-like contribution. The latter requires 9 $N_{\text{grid}} \times N_{\text{grid}}$ intermediates, namely the THC $\mathbf{Z}$-tensor, intermediates $\mathbf{A}$ and $\mathbf{B}$ from the precontraction step for both spin cases, as well as four matrices for the accumulation of results. This results in a rough memory requirement estimate of

$$
\begin{aligned}
M_{\text{est},3} &= \underbrace{N_{\text{grid}}^2}_{\mathbf{Z}} + \underbrace{2}_{\alpha/\beta} \times \underbrace{2 \times N_{\text{grid}}^2}_{\mathbf{A}/\mathbf{B}} + \underbrace{4 \times N_{\text{grid}}^2}_{\text{helper intermediates}} \times \underbrace{\frac{8\,\text{B}}{1024^3\,\frac{\text{B}}{\text{GB}}}}_{\to\text{GB}} \\
&= 9\,N_{\text{grid}}^2 \frac{8\,\text{B}}{1024^3\,\frac{\text{B}}{\text{GB}}}.
\end{aligned}
\tag{11}
$$

We note, that the various transformed, half-transformed, and untransformed collocation matrices are neglected in this estimate. For a reasonably sized basis set the relations $N_{\text{grid}} \approx 10\,N_{\text{bf}}$ and $N_{\text{bf}} \approx N_{\text{virt}} \gg N_{\text{occ}}$ hold, and therefore even the most memory demanding collocation matrix, i.e., X_μ^P of size $N_{\text{grid}} \times N_{\text{bf}} \approx \frac{1}{10} N_{\text{grid}}^2$ is only a tenth of the size of the intermediates discussed above.

7 Comparison of (Selected-nuclei) ω-RI-CDD-MP2 and THC-ω-RI-CDD-MP2

7.1 Asymptotic Scaling Comparison

In the main part of this publication we demonstrated the superiority of THC-ω-RI-CDD-MP2 compared to ω-RI-CDD-MP2 in terms of runtime for a selection of application inspired organic radicals. Vogler et al.[S3] proposed a selected-nuclei variant of ω-RI-CDD-MP2 to reduce the long computation times of the ω-RI-CDD-MP2 by making use of the locality of the perturbation. Since both, the selected-nuclei variant of ω-RI-CDD-MP2 and THC-ω-RI-CDD-MP2, were proposed to overcome the shortcomings of ω-RI-CDD-MP2, here, the three methods are compared in terms of their asymptotic scaling behavior for linear alkyl radicals up to 4000 basis functions with the cc-pVDZ basis set.

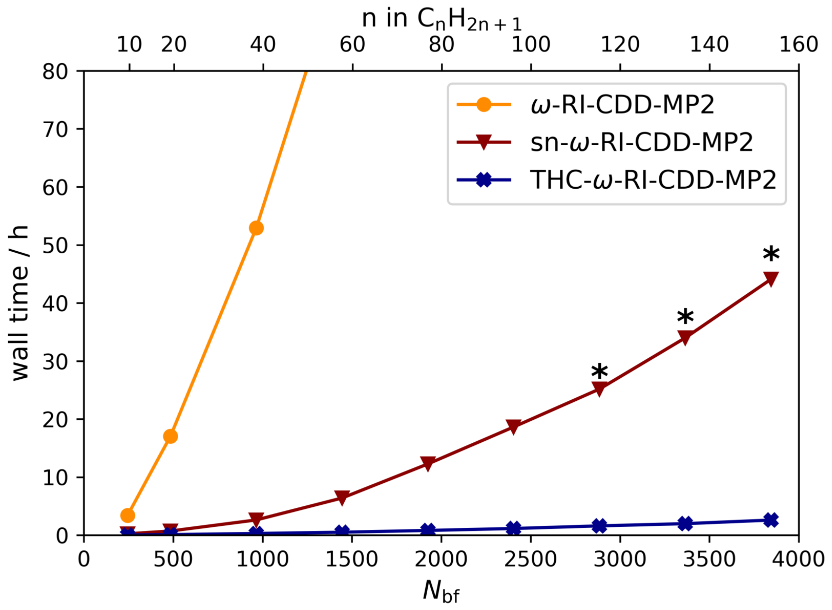

Figure S2: Comparison of the wall times and scaling of the ω-RI-CDD-MP2 (orange), the selected-nuclei ω-RI-CDD-MP2 (red), and the THC-ω-RI-CDD-MP2 method (blue) for linear alkyl radicals C_nH_{2n+1} (n ∈ {10, 20, 40, 60, 80, 100, 120, 140, 160}) using the cc-pVDZ basis set. Points marked with black asterisks were extrapolated conservatively.

As described in the literature,[S4] the RI-CDD-MP2 method is expected to scale quadratically

due to the dominant runtime contribution of the formation of the **R**-matrices. Here, even subquadratic scaling is observed, which is because here – unlike in the original publication – an attenuated Coulomb metric in the RI integrals was used. The selected-nuclei variant of ω-RI-CDD-MP2 improves the runtimes considerably with similar subquadratic scaling. More drastic improvements are however obtained with the THC-ω-RI-CDD-MP2 method, which, as demonstrated in the main part of the publication, also has effective asymptotic subquadratic scaling. For the largest alkyl chain computed with the ω-RI-CDD-MP2 method, the selected-nuclei version provides a speedup of a factor of 15, while the THC method achieves a speedup of close to 200. It is apparent, that while all methods share an asymptotic subquadratic scaling, the drastically smaller prefactor – in addition to the reduced memory requirements – of the THC-ω-RI-CDD-MP2 make it vastly superior in terms of runtime. Furthermore, in contrast to the selected-nuclei ω-RI-CDD-MP2 method, the THC method is not limited to selected nuclei only but provides HFCCs for all nuclei simultaneously at reduced cost. In principle, THC-factorized ERIs can also be applied in the selected-nuclei approach, potentially leading to further speedups.

7.2 Timing Comparison With Selected-nuclei ω-RI-CDD-MP2

To compare the THC-ω-RI-CDD-MP2 method against the selected-nuclei variant of ω-RI-CDD-MP2 for more chemically relevant molecules, Table 4 of the main part of this publication is extended with timings obtained with the selected-nuclei ω-RI-CDD-MP2 method (see Table S3).

While the selected-nuclei method significantly improves upon the ω-RI-CDD-MP2 implementation in terms of computation time, with speedups up to 30–70, the THC-based method considerably outperforms both with speedups up to roughly 600–1000. The computational savings for the selected-nuclei method are greatest if the molecule under investigation is predominantly one-dimensional, like $(\text{glu})_4$, whereas for globular systems, like the solvated TEMPO radical, speedups are reduced. The effect is also observed for the THC-ω-RI-CDD-

Table S3: Comparison of the memory requirements M_{est}, timings t, and relative speedups S of the THC-ω-RI-CDD-MP2 method with the ω-RI-CDD-MP2 method and its selected-nuclei variant. Speedups are determined relative to the timings obtained with the ω-RI-CDD-MP2 method.

			ω-RI-CDD-MP2		sel.-nuc. ω-RI-CDD-MP2			THC-ω-RI-CDD-MP2		
	molecule	N_{bf}	M_{est} / GB	t / h	M_{est} / GB	t / h	S	M_{est} / GB	t / h	S
cc-pVDZ	$C_{60}H_{121}$	1445	201.0	101.8	358.2	8.7	12	11.7	0.7	145
	TEMPO_{H_2O}	1444	208.5	345.9	366.0	51.1	7	11.7	1.5	230
	$(\mathrm{glu})_4$	1074	90.0	380.2	157.8	12.0	32	5.9	0.7	540
	PTMA_3	1102	93.8	620.9	165.8	18.1	34	6.5	1.2	517
	$(\mathrm{AT})_2$	1566	286.0	1111.0	500.3	53.1	21	23.1	1.9	585
cc-pVTZ	$C_{20}H_{41}$	1174	66.1	363.3	124.6	11.1	33	8.7	0.6	606
	TEMPO	582	8.2	160.1	15.5	3.4	47	2.1	0.2	891
	Tyr	530	6.6	99.4	12.3	2.2	45	1.4	0.1	780
	Thy	1260	86.6	1346.2[a)]	161.5	18.4	73	8.7	1.1	1224
	$(\mathrm{AT})_1$	1982	340.4	2727.8[a)]	634.0	38.1	72	20.7	3.1	880

[a)] timings are estimated conservatively based on the time taken for the first Laplace point

MP2 method, since the efficiency of both methods relies on the sparsity of the pseudo-density matrices. It is however less pronounced, indicating that the algorithm for the exchange-like part in the THC-ω-RI-CDD-MP2 utilizes the sparsity well even in nonlinear systems.

8 Structures

8.1 Benchmark Set of Small Organic Radicals

All structures were taken from the original publication by Vogler *et al.*[S2] and are available for download in a zip archive in the Supporting Information.

8.2 Benchmark Set of Medium-sized Organic Radicals

All structures used for optimizing the screening thresholds in the Coulomb- and exchange-like part of the **R**-matrices were optimized with the DL-FIND[S5] subprogram of FermiONs++[S6–S8] using the ωB97X functional and the def2-TZVP basis set. All structures are available for download in a zip archive in the Supporting Information. The benchmark set includes the following molecules and supramolecular assemblies:

- a linear alkyl radical: $C_{60}H_{121}$
- a spin-labeled adenine-thymine DNA fragment: $(AT)_2$
- a spin-labeled amylose chain: $(glu)_4$
- the poly(2,2,6,6-tetramethyl-piperidenyloxyl-4-yl methacrylate) (PTMA) trimer: $PTMA_3$
- a H_2O solvated (2,2,6,6-Tetramethylpiperidin-1-yl)oxyl (TEMPO) radical with 50 water molecules: $TEMPO_{H_2O}$
- a supramolecular assembly of the *tert*-butyl radical in a cucurbit[6]uril (CB[6]) host: *t*-butyl@CB[6]

8.3 Test Set for the Comparison Against ω-RI-CDD-MP2

All structures used for the comparison against ω-RI-CDD-MP2 were optimized with the DL-FIND[S5] subprogram of FermiONs++[S6–S8] using the ωB97X functional and the def2-TZVP basis set. All structures are available for download in a zip archive in the Supporting

Information.

The benchmark set includes the following molecules and supramolecular assemblies ...

- ... for the cc-pVDZ basis set:
  - a linear alkyl radical: $C_{60}H_{121}$
  - a H_2O solvated TEMPO radical with 50 water molecules: $TEMPO_{H_2O}$
  - a spin-labeled amylose chain: $(glu)_4$
  - the PTMA trimer: $PTMA_3$
  - a spin-labeled adenine-thymine DNA fragment: $(AT)_2$
- ... for the cc-pVTZ basis set:
  - a linear alkyl radical: $C_{20}H_{41}$
  - the TEMPO radical: TEMPO
  - the tyrosine radical: Tyr
  - a spin-labeled thymine molecule: Thy
  - a spin-labeled adenine-thymine DNA fragment: $(AT)_1$

References

(S1) Parrish, R. M.; Hohenstein, E. G.; Martínez, T. J.; Sherrill, C. D. Tensor hypercontraction. II. Least-squares renormalization. *J. Chem. Phys.* **2012**, *137*, 224106.

(S2) Vogler, S.; Dietschreit, J. C.; Peters, L. D.; Ochsenfeld, C. Important components for accurate hyperfine coupling constants: electron correlation, dynamic contributions, and solvation effects. *Mol. Phys.* **2020**, *118*.

(S3) Vogler, S.; Savasci, G.; Ludwig, M.; Ochsenfeld, C. Selected-Nuclei Method for the Computation of Hyperfine Coupling Constants within Second-Order Møller-Plesset Perturbation Theory. *J. Chem. Theory. Comput.* **2018**, *14*, 3014–3024.

(S4) Vogler, S.; Ludwig, M.; Maurer, M.; Ochsenfeld, C. Low-scaling first-order properties within second-order Møller-Plesset perturbation theory using Cholesky decomposed density matrices. *J. Chem. Phys.* **2017**, *147*, 024101.

(S5) Kästner, J.; Carr, J. M.; Keal, T. W.; Thiel, W.; Wander, A.; Sherwood, P. DL-FIND: An open-source geometry optimizer for atomistic simulations. *J. Chem. Phys. A* **2009**, *113*, 11856–11865.

(S6) Kussmann, J.; Ochsenfeld, C. Pre-selective screening for matrix elements in linear-scaling exact exchange calculations. *J. Chem. Phys.* **2013**, *138*, 134114.

(S7) Kussmann, J.; Ochsenfeld, C. Preselective screening for linear-scaling exact exchange-gradient calculations for graphics processing units and general strong-scaling massively parallel calculations. *J. Chem. Theory. Comput.* **2015**, *11*, 918–922.

(S8) Kussmann, J.; Ochsenfeld, C. Hybrid CPU/GPU Integral Engine for Strong-Scaling Ab Initio Methods. *J. Chem. Theory. Comput.* **2017**, *13*, 3153–3159.
